# Youth Perspectives on the Climate Crisis: Motivation and Action Pathways

**DOI:** 10.1111/desc.70217

**Published:** 2026-05-15

**Authors:** Anne‐Wil Kramer, Judith van de Wetering, Suzanne van de Groep, Lysanne W. te Brinke, Yara J. Toenders, Kayla H. Green, Eveline A. Crone

**Affiliations:** ^1^ Erasmus School of Social and Behavioral Sciences Erasmus University Rotterdam Rotterdam The Netherlands; ^2^ Behavioral Science Institute Radboud University Nijmegen The Netherlands; ^3^ Department of Psychology, Education and Child Studies, Erasmus School of Social and Behavioral Sciences Erasmus University Rotterdam Rotterdam The Netherlands; ^4^ Developmental and Educational Psychology Leiden University Leiden The Netherlands

**Keywords:** collective action, motivation, pro‐environmental behavior, wellbeing, worry about climate change, youth participatory research

## Abstract

Young people can be agents of change for urgently needed sustainable transitions. We combine survey data from the Urban Rotterdam Project (2021 sample: *N* = 1152, age range = 12–28 years, *M*_age_ = 19.07, *SD*_age_ = 2.87, 54% female, 16% bi‐ or multicultural background; 2023 sample: *N* = 327, age range = 13–28, *M*_age_ = 19.24, *SD*_age_ = 2.86, 67% female, 24% bi‐ or multicultural background) with youth participatory data (2024 sample: *N* = 114, age range = 16–26, *M*_age_ = 20, 51% female, 37% bi‐ or multicultural background) to examine youths’ climate‐related worry, affective wellbeing, motivations for pro‐environmental behavior and preferences for climate change communication. First, drawing on self‐determination theory, we tested whether associations between climate‐related worry, pro‐environmental behavior, and affective wellbeing depend on the quality of youths’ motivation to engage in pro‐environmental behavior. We found that climate‐related worry was associated with increased pro‐environmental behavior for youth who acted out of interest and enjoyment (i.e., intrinsic motivation) or internal pressure (i.e. introjected motivation). Youth who had internalized pro‐environmental behavior as personally important (i.e. identified motivation) showed high levels of pro‐environmental behavior regardless of worry. Climate‐related worry was also robustly associated with reduced affective wellbeing, irrespective of motivation type. Additionally, adopting a youth participatory approach, we explored youths’ preferences for climate change communication and pro‐environmental behaviors. We found that youth were more motivated by collective‐systemic than individual pro‐environmental actions because of benefits for motivation (“Alone it feels useless”), affective wellbeing (“Doing something together gives me hope”), and sustainability (“Cheaper public transport would make my choices matter”). We also found that youth preferred risk‐emphasizing, positive, and empowering communication strategies over approaches including humor and purely factual information. Overall, our research illustrates the importance of supporting youths' motivation to help them turn their climate‐related worry into pro‐environmental behavior, presents youths' perspectives on what motivating climate communication and action pathways might look like, and highlights the value of involving youth as active contributors to research and evidence‐informed climate action.

**Summary:**

- The present study combined survey and participatory methods to examine youths' climate‐related worry, affective wellbeing, motivations for pro‐environmental behavior and preferences for climate change communication.
- Climate‐related worry was more strongly linked to pro‐environmental behavior when youth were motivated by interest and enjoyment (intrinsic) or by internal pressure such as guilt (introjected), though introjected motivation may carry wellbeing costs.
- Youth who had internalized pro‐environmental behavior as personally important (identified motivation) engaged in more pro‐environmental behavior regardless of their worry levels.
- Climate‐related worry was robustly associated with reduced affective wellbeing, irrespective of motivation type.
- Youth preferred collective‐systemic over individual pro‐environmental behaviors, while also recognizing the interconnectedness between the two.
- Youth preferred climate communication that is direct, positive, and empowering.

## 1 Youth Perspectives on the Climate Crisis: Motivation and Preferred Mitigation Pathways

Today's young generation is projected to experience unprecedented consequences of climate change, including heatwaves, droughts, and floods (Thiery et al. 2021; Sanson et al. 2019, UNICEF 2021). Because many youth feel worried about climate change (Hickman et al. 2021; Poortinga et al. 2019; UNDP 2021) and are motivated to contribute to society (Fuligni 2019), they are increasingly viewed as agents of change for urgently needed sustainable shifts (Bandura and Cherry 2020; Crone et al. 2024; Thomaes et al. 2023). In line with this view, many interventions, most of them educational, have been developed to promote youth's pro‐environmental behavior (Świątkowski et al. 2024; Van De Wetering et al. 2022). A common assumption across these approaches is that climate‐related worry can motivate engagement. But climate‐related worry can be a double‐edged sword: it may promote pro‐environmental behavior, but it is also associated with lower wellbeing (Becht et al. 2024; Ogunbode et al. 2022; Ojala 2023; Ramadan et al. 2023; Veijonaho et al. 2025; Wullenkord et al. 2025). This makes it important to understand when climate‐related worry motivates youth's pro‐environmental behavior and when it diminishes their wellbeing, as well as which intervention approaches youth perceive as motivating for pro‐environmental behavior. To address this, the present study (a) draws on self‐determination theory to test whether associations between climate‐related worry and pro‐environmental behavior, as well as between climate‐related worry and affective wellbeing, depend on the quality and reasons underlying youth's motivation to engage in pro‐environmental behavior; and (b) adopts a youth participatory approach to explore youth's preferences for climate change communication and the types of pro‐environmental behaviors they are motivated to engage in.

### 1.1 Youth's Climate‐Related Worry and Pro‐Environmental Behavior

We broadly define youth as adolescents and emerging adults aged 12 to 30 years old (Sawyer et al. 2018). This currently is the generation that experiences relatively high levels of climate‐related worry: In a global survey, the majority of youth agreed that “the future is frightening” and that “humanity is doomed” because of climate change (Hickman et al. 2021). Youth are at the forefront of the climate movement (Thomaes 2026). For example, Greta Thunberg's 2019 school strikes mobilized millions of youth to participate in Fridays for Future marches, legal action, and public debate (de Moor et al. 2020; Neas et al. 2022; Wahlström et al. 2020). Climate activism is just one way in which youth can transform their climate‐related worry into action. They can engage in a variety of pro‐environmental behaviors, ranging from individual private behaviors (e.g. changing dietary, transport, and consumption habits; and conserving water and energy) to more collective‐systemic public behaviors (e.g. petitioning, donating, and voting; Stern 2000; Hampton and Whitmarsh 2023).

Climate anxiety and distress are typically associated with pathology and functional impairment. However, as youths’ concerns about climate change are not necessarily pathological or irrational (Becht et al. 2024; Sanson and Bellemo 2021), we here focus on climate‐related worry, which can be defined as recurrent, difficult‐to‐control thoughts about climate change and its potential negative consequences, and hence represents the cognitive aspect of climate anxiety (Donati et al. 2025). Climate‐related worry is positively associated with pro‐environmental behavior across countries and cultural contexts (Ogunbode et al. 2022). Yet developmental studies show that not all youth are able to transform their worry into action (Krettenauer et al. 2020; Olsson et al. 2016): for some, climate‐related worry negatively impacts their wellbeing (Hickman et al. 2021; Veijonaho et al. 2024; Wullenkord et al. 2023). Youth may be especially vulnerable to the impacts of climate‐related worry on wellbeing, given their heightened vulnerability to mood disorders (Solmi et al. 2022). Recent work suggests that climate‐related worry is more likely to translate into pro‐environmental behavior when youth are worried about consequences for others, such as future generations or non‐human species, rather than only for themselves or close others (Wullenkord et al. 2023); when they perceive pro‐environmental behavior as effective (Becht et al. 2024; Qin et al. 2024; Sachisthal et al. 2025); and when they use adaptive emotion regulation and coping strategies (Veijonaho et al. 2025; Wullenkord et al. 2025). This research thus suggests that climate‐related worry may foster pro‐environmental behavior among youth if accompanied by opportunities to act in ways that feel effective, autonomous, and socially embedded.

### 1.2 A Self‐Determination Theory Lens to Motivation for Pro‐Environmental Behavior

We build on prior work (Spitzer et al. 2024) to argue that self‐determination theory offers an overarching framework to understand when climate‐related worry is more likely to foster youth's pro‐environmental behavior, and when it is more likely to diminish youth's wellbeing. Self‐determination theory proposes that people have basic psychological needs for autonomy, competence, and relatedness, and that the satisfaction of these needs shapes the quality of motivation with which they engage in behaviors (Ryan and Deci 2000; Ryan and Deci 2020). We conceptualize motivation as the process that invigorates and sustains goal‐directed behavior (Schunk et al. 2014). Importantly, motivation is situated in context and can fluctuate as a function of contextual demands and supports (Vu et al. 2022). This implies that interventions and communication approaches can shape motivational quality, for example, by supporting or undermining autonomy, competence, and relatedness (Vu et al. 2022; Kramer et al. 2024; Kramer et al. 2021). From this perspective, climate‐related worry may be more likely to foster youth's pro‐environmental behavior when youth experience need satisfaction. In that case, youth can engage in pro‐environmental behavior as volitional, personally meaningful, and socially connected, which is typically linked to higher wellbeing (Ryan and Deci 2020; Martela and Sheldon 2019; Ryan et al. 2022; Spitzer et al. 2024). Conversely, when youth experience lower need satisfaction, climate‐related worry may be more likely to undermine youth's wellbeing, for example, by fueling pressure, guilt, or helplessness.

A key distinction in self‐determination theory is between autonomous motivation (i.e. intrinsic and identified) and controlled motivation (i.e. introjected and extrinsic) (Ryan and Deci 2000). Intrinsic motivation refers to acting because the activity is enjoyable or interesting, whereas identified motivation refers to acting because it aligns with one's values and goals. In contrast, introjected and extrinsic motivation reflect acting from internal or external pressure, such as internal guilt or shame (i.e. introjected) or to obtain external rewards or avoid punishment (i.e. extrinsic). Across domains, autonomous motivation predicts more sustained engagement and higher wellbeing, whereas controlled motivation is more often linked to more fragile engagement and lower wellbeing, particularly when goals are demanding (Kramer et al. 2023, Kramer et al. 2025; Milyavskaya et al. 2015; Ng et al. 2012; Vansteenkiste et al. 2004). In environmental contexts, autonomous motivation has been associated with more pro‐environmental behavior (Pelletier et al. 1998; Renaud‐Dubé et al. 2010; van der Werff et al. 2013) and higher affective wellbeing (Spitzer et al. 2023), and similar patterns emerge when youth perceive pro‐environmental behavior as compatible with their values and identity (Grapsas et al. 2023).

Taken together, self‐determination theory helps specify why climate‐related worry can be a double‐edged sword. When climate‐related worry is coupled with more autonomous motivation, youth may be more likely to translate climate‐related worry into pro‐environmental behavior while maintaining higher wellbeing. When climate‐related worry is coupled with more controlled motivation, youth may be more likely to experience lower wellbeing, and pro‐environmental behavior may be less sustained.

### 1.3 Youths’ Preferences for Climate Change Communication and Mitigation

If individual differences in motivation to engage in pro‐environmental behavior determine whether climate‐related worry is channeled into action, it then becomes crucial to understand youths’ preferences for how to be informed about and involved in pro‐environmental behavior. Many interventions and communication strategies have been developed to capitalize on youth's potential as change agents and promote their pro‐environmental behavior (Ardoin et al. 2018; Świątkowski et al. 2024; Van De Wetering et al. 2022). These existing interventions make use of diverse social influence (e.g. role modeling, social norm setting) and educational (e.g. lectures, field trips, hands‐on investigation) strategies and are moderately effective at promoting pro‐environmental behavior among youth (Ardoin et al. 2018; Świątkowski et al. 2024; Van De Wetering et al. 2022). However, the effectiveness of existing interventions has not increased over the past decades and tends to be lower among older youth (Świątkowski et al. 2024; Van De Wetering et al. 2022; Vlasceanu et al. 2024). To further optimize interventions that promote youth's pro‐environmental behavior, we need to understand the communication strategies that youth prefer and the types of pro‐environmental behavior that they perceive as important for climate change mitigation.

In the current study, we therefore explore youth's preferences for climate communication strategies. From a rights perspective, youth have the right to be heard and adequately informed on issues that directly affect them, including climate change (United Nations 1989; UNICEF 2021). However, youth are typically less satisfied with their climate change education than younger children (UNESCO 2022), suggesting that existing communication strategies are not well‐aligned with their needs (Thomaes et al. 2023). For example, while fear‐based communication can capture attention, it may undermine motivation if not combined with effective pathways for action (Spitzer et al. 2024). Conversely, purely positive messages may be experienced as downplaying the seriousness of the problem, while information heavy messages that communicate what youth already know may be experienced as infantilizing, thereby demotivating action (Nabi and Myrick 2019; Ojala 2022; O'Neill and Nicholson‐Cole 2009; Yeager et al. 2018).

Youths’ perspectives are also valuable in selecting pro‐environmental behaviors for interventions to target. Youth can contribute to climate change mitigation in many ways (Hampton and Whitmarsh 2023), yet they may prefer some types of pro‐environmental behaviors over others. That is, youth may perceive some behaviors to be more efficacious or rewarding than others (Spitzer et al. 2024). For example, a recent study found that autonomy‐supportive climate change communication strategies motivated youths’ petitioning but not donating behavior, presumably because petitioning is more directly aligned with youth's motivation to stand up for social justice (van de Wetering et al. 2025). Another recent study suggested that many youth feel that reducing their everyday meat consumption is not realistic, as their parents mostly decide what they eat, so they experience no autonomy in this choice (van de Wetering et al. 2025). From a self‐determination theory perspective, youth's preferences for particular types of pro‐environmental behavior may partly reflect how well those behaviors satisfy their basic psychological needs. Behaviors that feel autonomously chosen, that allow youth to feel competent and effective, and that are embedded in social connections may be experienced as more motivating than behaviors that feel imposed, ineffective, or individual (Ryan and Deci 2020; Spitzer et al. 2024). We therefore argue that youth can, from their lived experiences, propose concrete pro‐environmental behaviors to help mitigate climate change at home, in school, and in their communities (Crone et al. 2024; Toenders et al. 2024). Youth also have lived experience on what support they may need to engage in those behaviors. In the current study, we explore what kinds of pro‐environmental behaviors and structural supports young people themselves view as important, feasible and motivating.

Taken together, besides our survey study, we adopted a youth participatory approach, actively engaging young people in identifying underlying problems and co‐creating solutions for the climate crisis that matter to them (Ozer et al. 2024). Our insights can directly inform the design of interventions that fit youths’ lived experiences and have the potential to foster more autonomous motivation for pro‐environmental behavior. By positioning youth not only as research subjects but also as partners, participatory approaches also increase the relevance and legitimacy of climate recommendations, while strengthening youths’ sense of agency and critical consciousness (Anyon et al. 2018; Toenders et al. 2024).

### 1.4 The Present Study

The present study combines survey data with participatory insights to address two main goals. First, we test whether the associations between climate‐related worry and pro‐environmental behavior, as well as between climate‐related worry and affective wellbeing depend on the quality of youths’ motivation. Second, we explore youths’ perspectives on what feels motivating in practice, focusing on their preferences for climate change communication and for individual versus collective, systemic pro‐environmental behaviors. Together, this evidence informs how interventions can create motivational contexts that help youth turn climate‐related worry into meaningful and sustainable engagement, thereby contributing to Sustainable Development Goals to take action to combat climate change in a way that supports inclusive institutions and education, ultimately contributing to youths’ wellbeing (SDGs 13, 16, 4, and 3, respectively).

We draw on data from two complementary Urban Rotterdam studies: a survey study conducted within the Urban Rotterdam Project (https://doi.org/10.17605/OSF.IO/H5X2A) and a participatory study via our YoungXperts platform (https://www.youngxperts.nl/). Both studies aimed to oversample groups that are typically underrepresented in environmental and social science research. Existing work predominantly focuses on highly engaged, pre‐university educated, and monocultural youth (Sachisthal et al. 2025). Yet vocationally educated and bi‐ or multicultural youth may hold perspectives and face barriers that are crucial for developing equitable climate change solutions.

## 2 Method

### 2.1 Study Design

This project used a sequential design with two connected phases: a survey study followed by a participatory youth panel study. First, we analyzed survey data to map key patterns in youths' climate‐related worry, wellbeing, pro‐environmental behavior, and preferences for climate communication and action. We then brought these main survey findings back to youth in 11 interactive YoungXperts sessions, where participants discussed the results in plain language, added lived experience and context, and translated them into priorities and concrete “take‐actions”: actionable steps for people, organizations, companies, and government bundled in a manifesto. This manifesto was presented, together with youth, to the Dutch minister for Climate and Energy.

Because youth participation started after the survey phase, this was not a full Youth Participatory Action Research cycle across the entire research process. Instead, participatory methods were embedded in the later stages through co‐interpretation and co‐design of recommendations. This sequential approach allowed us to combine broad survey evidence with adolescents' lived experiences when formulating evidence informed recommendations, a key advantage noted by Toenders et al. (2024).

### 2.2 Survey Study

#### 2.2.1 Participants

Survey data were collected through the Urban Rotterdam Project, a study on youth development and societal engagement in the greater Rotterdam region in the Netherlands (project registration: https://doi.org/10.17605/OSF.IO/H5X2A). This project consists of nine biannual waves. Here, we separately analyzed data from two waves in which different data sets were collected. This study mostly made use of the data collected in December 2023 among 327 youth (see Table 1 for demographic information). In addition, for one measure (i.e. pro‐environmental behavior preference), we reanalyzed (te Brinke & Crone, *manuscript under review*) the data collected in December 2021 among 1152 youth (see Table 1 for demographic information), because this measure served as the main input for the youth participatory sessions.

**TABLE 1:** Demographic information of study samples.

Variable		Survey study		Participatory study
		2023 sample	2021 sample	2024 sample
Total *N*		327	1152	114
**Age**	Range	13–28	12–28	16–26
	*M* (*SD*)	19.24 (2.86)	19.07 (2.87)	20
**Gender**	Male	97 (29.7%)	386 (33.5%)	53 (46.5%)
	Female	219 (67.0%)	621 (53.9%)	58 (50.9%)
	Non‐binary	9 (2.8%)	26 (2.3%)	3 (2.6%)
	Prefer not to say/unknown	2 (0.6%)	118 (10.2%)	2 (1.8%)
**Education**	(Pre)‐vocational	48 (14.7%)	629 (54.6%)	54 (47.4%)
	(Pre)‐applied university	88 (26.9%)	128 (11.1%)	14 (12.3%)
	(Pre)‐university	142 (43.4%)	392 (34%)	43 (37.7%)
	Other (working)	49 (15.0%)	5 (0.4%)	3 (2.6%)
**Cultural background**	Dutch only	226 (69.1%)	968 (84%)	72 (63.2%)
	Non‐Dutch only	21 (6.4%)	61 (5.3%)	3 (2.6%)
	Dutch and other	69 (21.1%)	99 (8.6%)	31 (27.2%)
	Non‐Dutch and other	11 (3.4%)	23 (2.1%)	7 (6.1%)

*Note*: Dutch only = exclusively Dutch background; Non‐Dutch only = a single non‐Dutch background; Dutch and other = Dutch combined with one or more other backgrounds; Non‐Dutch and other = two or more non‐Dutch backgrounds.

Participants were recruited via educational institutions and local youth networks, with an emphasis on including youth from (pre‐)vocational educational tracks and multicultural backgrounds who are often underrepresented in scientific research (Green et al. 2023). Surveys were administered digitally. Participation was voluntary and all participants (and parents of participants younger than 16) provided informed consent. Participants received a 10‐euro compensation for participation per wave. The study was approved by the institutional ethical review board of the Psychology Department of the Erasmus University Rotterdam.

#### 2.2.2 Measures

An overview of survey measures is presented in Table 2.

**TABLE 2:** Overview of survey measures.

Measure	Survey sample	Number of items	Possible range	Observed range	*M*	*SD*	Alpha
Climate‐related worry	2023	10	10–50	10–50	25.35	10.54	0.95
Affective wellbeing	2023	19	14–70	14–67	51.98	11.24	0.94
Pro‐environmental behavior	2023	1	1–5	1–5	3.01	0.99	n.a.
Pro‐environmental motivation: intrinsic	2023	4	1–5	1–5	2.84	1.11	0.92
Pro‐environmental motivation: identified	2023	4	1–5	1–5	3.33	1.15	0.92
Pro‐environmental motivation: introjected	2023	4	1–5	1–5	2.65	1.01	0.83
Pro‐environmental motivation: extrinsic	2023	4	1–5	1–5	2.17	0.86	0.81
Climate communication strategy preference	2023	6	1–6^a^	n.a.	n.a.	n.a.	n.a.
Pro‐environmental behavior preference	2021	1	−1 to 6^b^	n.a.	n.a.	n.a.	n.a.

*Note*: The range represents the minimum and maximum observed scores. Alpha is Cronbach's alpha. N.a. = not applicable as these variables are non‐continuous.

^a^Rated per strategy.

^b^Pro‐environmental behavior preference: originally six response options; recoded into a single index (−1 = individual > collective‐systemic, 0 = equal, 1 = collective‐systemic > individual).

##### 2.2.2.1 Climate‐Related Worry

We assessed climate‐related worry using the 10‐item Climate Change Worry Scale (CCWS; Stewart 2021; see also Donati et al. 2025), which taps into the cognitive component of climate anxiety. Items reflect verbal‐linguistic thoughts about changes that might occur in the climate system and its possible negative effects (e.g. “I worry about how climate change will affect future generations”) and difficulty controlling these thoughts (e.g. “Once I begin to worry about climate change, I find it difficult to stop”). Items were rated along a five‐point Likert scale (1 = *not agree at all*, to 5 = *fully agree*). A sum score was computed, with higher scores reflecting more worry about climate change.

##### 2.2.2.2 Pro‐Environmental Behavior

Pro‐environmental behavior was measured with a single self‐report item. Before answering, participants read an instruction defining climate‐beneficial behaviors and providing examples (“The following questions are about behaviors that are beneficial for the climate [for example: separating waste, eating vegetarian, taking short showers, participating in a climate protest, etc.].”). Participants then rated the item “I do things that benefit the climate” on a five‐point Likert scale (1 = never to 5 = very often).

##### 2.2.2.3 Affective wellbeing

We assessed one aspect of wellbeing, namely affective wellbeing (or mood). We used the 19‐item Profile of Mood States scale (POMS; Wald and Mellenbergh 1990), which asks participants to indicate to what extent they feel that a range of mood descriptions (e.g. nervousness) represent their current mood state. Items were rated along a five‐point Likert scale (1 = *not at all* to 5 = *extremely*). Following standard scoring procedures, five subscales reflecting negative mood (tension, depression, anger, fatigue, confusion) were combined into a single negative mood index, and the vigor subscale was treated as the sole positive mood index. Vigor reflects positive activation and energy, an example item includes feeling “energetic”. A Total Mood Disturbance (TMD) score was computed by summing all negative mood items and subtracting the sum of positive mood items, with higher TMD scores indicating lower affective wellbeing. For ease of interpretation, an affective wellbeing score was calculated by reversing the TMD score, such that higher values reflect greater affective wellbeing.

##### 2.2.2.4 Pro‐Environmental Motivation

We assessed pro‐environmental motivation using a 16‐item adaptation of the Self‐Regulation Questionnaire‐Academic (SRQ‐A; Vansteenkiste et al. 2009), tapping into four different types of motivation (see Table S4). *Intrinsic motivation* refers to engaging in pro‐environmental behavior because it is inherently enjoyable or interesting (e.g. “I do things that are good for the environment because I find them fun to do”). *Identified motivation* refers to engaging in such behavior because it is personally important and aligns with one's values (e.g. “I do things that are good for the environment because I think it is important”). *Introjected motivation* refers to acting in order to avoid guilt or anxiety, or to attain feelings of self‐worth (e.g. “I do things that are good for the environment because I would feel bad about myself if I did not”). *Extrinsic motivation* refers to engaging in pro‐environmental behavior due to external demands, rewards, or punishments (e.g. “I do things that are good for the environment because others [i.e. parents, friends, teachers] make me do it”). Items were rated along a five‐point Likert scale (1 = *not at all true* to 5 = *very true*). A mean scale score was computed for each motivation type, with higher scores reflecting stronger endorsement of that type of motivation.

##### 2.2.2.5 Climate Communication Strategy Preference

To assess what climate communication strategies participants preferred, we designed six different communication messages using humor (“If we don't fight climate change, we can decorate palm trees for Christmas”), information (“Eating Big Macs with the whole class is just as bad for the climate as flying”), fear (“If the sea rises by 2 meters in the next 100 years due to climate change, the Netherlands will disappear under water”), social norm setting (“81% of young people are already taking action for the climate—are you?”), empowerment (“Young people are the future; together we can make sure our planet stays livable”), or positivity (“Taking action for the climate means you'll still be able to go snorkeling later in life”). Participants were asked: “The following questions are about how you respond to messages about climate change. How willing are you to take action against climate change after reading these statements?” After each message, participants rated their willingness on a five‐point Likert scale (1 = *not willing at all*, 5 = *extremely willing*).

We selected these six message strategies to represent theoretically and empirically distinct approaches that are widely discussed in the climate communication and behavior change literature (Suldovsky 2017). Specifically, informational messages reflect information deficit style communication that aims to increase knowledge; fear based messages foreground risks and potential negative consequences, with effectiveness depending in part on whether people also feel capable of responding; social norm messages draw on descriptive norms and social influence processes; empowerment messages emphasize agency and (collective) efficacy; positive messages use gain framing by highlighting desirable future outcomes; and humor is an affective strategy that can lower defensiveness and invite engagement with a potentially threatening topic (Boykoff and Osnes 2019). Together, these categories span both cognitive and affective pathways through which climate messages may shape willingness to act, while remaining sufficiently distinct to enable meaningful comparisons across strategies.

##### 2.2.2.6 Pro‐Environmental Behavior Preference

Participants were asked what pro‐environmental actions their generation finds important after answering whether they found combating climate change important. Participants who indicated that they found combatting climate change important (79.43% of total sample, *N* = 915) were then asked what they thought was the most important thing to do about climate change using a single item: “You think it's important to combat climate change. What should change to achieve this? You can select multiple answers”. We offered a list of six options based on youth participatory sessions (age range 16–23 years; as described in te Brinke & Crone, *manuscript under review*). Participants could select multiple responses. To explore preferences for pro‐environmental behaviors, we recoded participants’ selected preferences into two categories: collective‐systemic (“impose more rules for companies”, “produce more green energy”, “create more space for nature”) and individual (“eat fewer animal products”, “fly less”, “buy fewer things”). We calculated an index score by summing the item scores within each category, then subtracting the individual total from the collective‐systemic total. The resulting variable was coded as 1 when the collective‐systemic score exceeded the individual score, 0 when they were equal, and −1 when the individual score was higher.

### 2.3 Participatory Study

#### 2.3.1 Participants

Participatory data were gathered through 11 interactive focus groups conducted between March 2024 and June 2024 via the *YoungXperts* (http://youngxperts.nl) youth participation platform. These sessions involved a total of 114 youth (see Table 1 for demographic information) and were held at schools, community centers, and youth organizations across the Netherlands in groups of 5 to 15 youth. Recruitment was conducted through our collaboration partners (e.g. youth workers and educators), who invited young people within their existing networks to participate. As a result, the exact recruitment procedure varied by location and organizational context. Participants were informed in advance that the session would focus on climate‐related topics. Focus groups were not recorded but documented through meeting logs and archiving of session materials (e.g. sticky notes, flip over sheets). Participants received a 10‐euro compensation for participation. The study was approved by the institutional ethical review board of the Erasmus University Rotterdam.

#### 2.3.2 Procedure

Each focus group was co‐led by a researcher and, when applicable, by a youth worker, teacher or other supervisor, and took approximately 1 h. First, the facilitators presented so‐called FACTS about climate change and youth: plain‐language findings from the Urban Rotterdam Project survey data. For example, “Youth talk about climate with parents (63.9%) and friends (66.1%) but less so at school (39.3%)” (see Table S3 for more details). Next, participants engaged in a range of activities. In a brainstorm writing exercise, participants individually wrote about their desired future, barriers, motivators, and support needs on sticky notes. In a clustering exercise, facilitators worked with participants to group similar sticky notes together on a joint *future wall*. Once the notes were clustered and labeled, participants voted on the themes they considered most important, and, in a final discussion round, turned these priorities into concrete so called “take‐actions”: actionable steps that people, organizations, companies, and governments could take. By placing challenges and solutions side by side on the *future wall*, the sessions revealed how youth link their concerns to practical strategies, offering direct insight into the support and skills they believe are essential for sustained pro‐environmental engagement.

### 2.4 Data Analysis

#### 2.4.1 Moderation of the Association Between Climate‐Related Worry and Pro‐Environmental Behavior and Affective Wellbeing by Motivation

Survey data were analyzed using R version 4.5.1 (R core team 2024). For each scale, we computed mean scale scores. We standardized all continuous variables and treated all categorical variables as factors. We tested all effects against *α* = 0.05. We computed descriptives for all study variables and Pearson correlations among the continuous study variables.

First, to test to what extent the association between climate‐related worry and pro‐environmental behavior depends on motivation, we computed mean scores for intrinsic, identified, introjected, and extrinsic motivation, and conducted a multiple regression analysis with pro‐environmental behavior as the dependent variable. The model included climate‐related worry, the four motivation types and climate‐related worry x motivation type interaction terms. We also included age, educational level, gender, and cultural background as covariates, as prior research shows that these factors impact climate‐related worry and pro‐environmental behavior (Poortinga et al. 2019). For example, younger individuals tend to report higher levels of concern about climate change and pro‐environmental behavior (Krettenauer et al. 2020; Lee et al. 2020).

Second, we tested whether the relationship between climate‐related worry and affective wellbeing was moderated by motivation type. We fitted the same model, yet now the dependent variable was affective wellbeing instead of pro‐environmental behavior.

#### 2.4.2 Youth Preferences for Climate Change Communication and Mitigation

Next, to explore what communication strategies and actions youth preferred to address climate change, we conducted pairwise comparisons of mean ratings across communication strategies. We did the same for pro‐environmental behavior preferences.

Qualitative data from the focus groups were analyzed using affinity mapping, a collaborative and inductive technique for grouping related data points. This method allowed us to organize the focus group outputs (i.e. post its and flip overs) into coherent thematic clusters (Holtzblatt and Beyer 2017) and to iteratively refine these clusters into themes relevant to our research questions (Braun and Clarke 2006, 2019; Terry et al. 2017). For example, we created clusters of youth‐defined action strategies, perceived barriers to engagement, and recommendations for systemic action. We did not quantify themes (e.g. “*n* participants”), because our analytic aim was interpretive rather than prevalence focused, and we did not perform systematic frequency coding (Braun and Clarke 2019; Levitt et al. 2018; Sandelowski 2001). Instead, we substantiate each theme with illustrative extracts and describe converging and diverging perspectives (Levitt et al. 2018). We report the qualitative findings in the third part of the Results section, alongside quantitative results on preferred communication strategies and actions.

## 3 Results

### 3.1 Survey Study Results

Table 3 presents Pearson correlations among the survey study variables, showing that youth with higher levels of climate‐related worry engage in more pro‐environmental behavior and experience reduced affective wellbeing. Moreover, youth who engage in more pro‐environmental behavior report higher levels of intrinsic, identified, and introjected (but not extrinsic) motivation. Youth who experience higher affective wellbeing report lower levels of introjected and extrinsic (but not intrinsic or identified) motivation. Youth were most motivated to engage in pro‐environmental behavior by identified motives, followed by intrinsic and introjected motives, while extrinsic motives were reported as least motivating (see Table S2 for paired samples *t*‐tests).

**TABLE 3:** Correlations between survey study variables.

	1	2	3	4	5	6	7	8
1. Climate‐related worry	1							
2. Pro‐environmental behavior	0.39^***^	1						
3. Affective wellbeing	−0.20^***^	−0.06	1					
4. Intrinsic motivation	0.69^***^	0.51^***^	−0.06	1				
5. Identified motivation	0.72^***^	0.59^***^	−0.10	0.80^***^	1			
6. Introjected motivation	0.57^***^	0.30^***^	−0.19^***^	0.51^***^	0.61^***^	1		
7. Extrinsic motivation	0.17^**^	0.02	−0.11^*^	0.16^**^	0.13^*^	0.48^***^	1	
8. Age	0.06	0.08	−0.02	0.03	0.10	0.15^**^	0.03	1

^***^ *p* < 0.001, ^**^ *p* < 0.01, ^*^ *p* < 0.05.

#### 3.1.1 Pro‐Environmental Behavior: Moderation of the Association With Climate‐Related Worry by Motivation

We found no main effect of climate‐related worry on pro‐environmental behavior (*β* = −0.045, *p* = 0.568), indicating that climate‐related worry was not associated with pro‐environmental behavior at average levels of all motivation types. We found a positive main effect of identified motivation on pro‐environmental behavior (*β* = 0.535, *p* < 0.001), but no main effects of intrinsic (*β* = 0.108, *p* = 0.221), introjected (*β* = −0.103, *p* = 0.169), and extrinsic (*β* = −0.009, *p* = 0.890) motivation. Covariates were not significantly associated with pro‐environmental behavior in our model. Because these are standardized coefficients, the pattern suggests a comparatively strong association for identified motivation, while the other main effects were small in magnitude.

**FIGURE 1:**
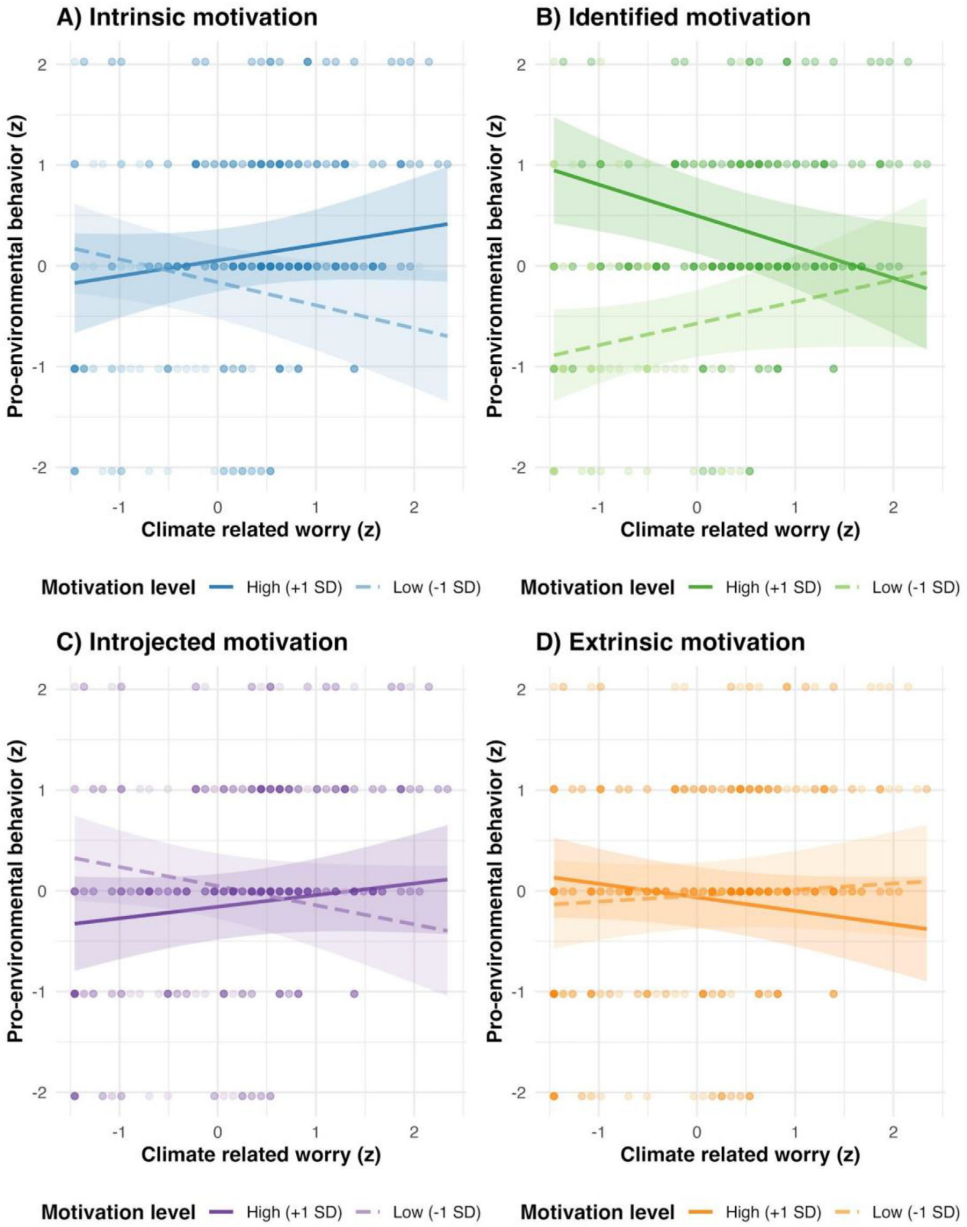
Motivation type moderates the association between climate‐related worry and pro‐environmental behavior. Shaded areas represent +/− SEM (95% CI).

Importantly, we did find that motivation type moderated the association between climate‐related worry and pro‐environmental behavior. These interactions are visualized in Figure 1. Specifically, the association was more positive at higher levels of intrinsic motivation (*β* = 0.191, *p* = 0.015) and introjected motivation (*β* = 0.153, *p* = 0.038). In contrast, identified motivation significantly attenuated the worry‐behavior association (*β* = −0.263, *p* = 0.006). The interaction between worry and extrinsic motivation was negative but not statistically significant (*β* = −0.097, *p* = 0.072). Overall, the interaction effects were small to moderate in standardized terms.

Overall, climate‐related worry was not directly associated with pro‐environmental behavior, whereas identified motivation showed a strong positive association with pro‐environmental behavior. However, the worry‐behavior link depended on motivational quality: it was more positive at higher intrinsic and introjected motivation, attenuated at higher identified motivation, and not reliably moderated by extrinsic motivation.

#### 3.1.2 Affective Wellbeing: Moderation of the Association With Climate‐Related Worry by Motivation

We found a negative main effect of climate‐related worry on affective wellbeing (*β* = −0.248, *p* = 0.010), indicating that higher worry was related to reduced affective wellbeing. We also found a positive main effect of intrinsic motivation on affective wellbeing (*β* = 0.241, *p* = 0.025), but no main effects of extrinsic (*β* = −0.075, *p* = 0.325), identified (*β* = −0.115, *p* = 0.354), and introjected (*β* = −0.088, *p* = 0.327) motivation. The effects of worry and intrinsic motivation were modest in magnitude, whereas the remaining motivation effects were small. Some covariates were significantly associated with affective wellbeing in our model: a higher educational level was positively associated with affective wellbeing (medium: *β* = 0.387, *p* = 0.034; high: *β* = 0.422, *p* = 0.017), and female gender was negatively associated with affective wellbeing (compared to the male reference category: *β* = −0.265, *p* = 0.046; non‐binary youth: *β* = −0.522, *p* = 0.222; youth preferring not to answer: *β* = 1.015, *p* = 0.124).

Importantly, we did not find that motivation type moderated the association between climate‐related worry and affective wellbeing (intrinsic: *β* = −0.036, *p* = 0.710; extrinsic: *β* = 0.037, *p* = 0.570; identified: *β* = −0.017, *p* = 0.884; introjected: *β* = 0.001, *p* = 0.990).

Together, this indicates that climate‐related worry is robustly linked to poorer affective wellbeing, while intrinsic motivation to engage in pro‐environmental behavior, as well as higher educational level, might serve as protective factors associated with better affective wellbeing. However, motivation type does not appear to buffer (or exacerbate) the affective wellbeing costs of worry.

### 3.2 Youth Preferences for Climate Change Communication and Mitigation

The survey analyses quantified how climate‐related worry, motivation, pro‐environmental behavior, and affective wellbeing were related, but they cannot explain how youth interpret climate change communication or why particular communication approaches and behaviors feel motivating. We therefore complemented the survey with participatory focus groups to contextualize the survey patterns, with prompts informed by participants’ survey responses (Table S3). Table 4 presents an overview of the thematic clusters that resulted from the affinity mapping of the focus group data, after which we describe these patterns in more detail.

**TABLE 4:** Overview of data clusters resulting from affinity mapping of the focus groups.

Cluster	Description	Illustrative quote
1. Emotional burden and coping	A fear of irreversible change, sleep loss, but also peer reassurance	“Climate videos keep me awake; talking with friends helps.”
2. Motivation pathways	A sense of collective efficacy, pressure of social‐norms, and identity‐based action	“If my friends join a clean‐up, I join too; alone it feels useless.”
3. Shared responsibility across levels	Interconnectedness of individual habits, collective activism, and supportive policy	“Cheaper public transport, not cheaper flights, would make my choices matter.”
4. Mental‐health scaffolds	Experienced hope through joint action, boosted mood through community service	“Doing something together gives me hope.”
5. Climate education and skills	Mandatory cross‐curricular climate education, and local solution labs	“We learn the science but not how to fix it or talk to people with different opinions, yet sometimes, we also don't even learn about the real facts; I didn't know it was this bad.”
6. Motivation levers and barriers	The potential of financial incentives, gamified challenges, and role model messaging	“Make plant‐based food cheaper and show when the government or celebrities do something good for the climate.”
7. Youth voice in decision‐making	Lowering the voting age to 16, and setting up youth citizens’ assemblies and generational tests for laws	“Nothing about us without us. Being taken seriously leads to taking things seriously.”

#### 3.2.1 Youth Prefer Fear‐Based, Positive, and Empowering Climate Communication Strategies

**FIGURE 2:**
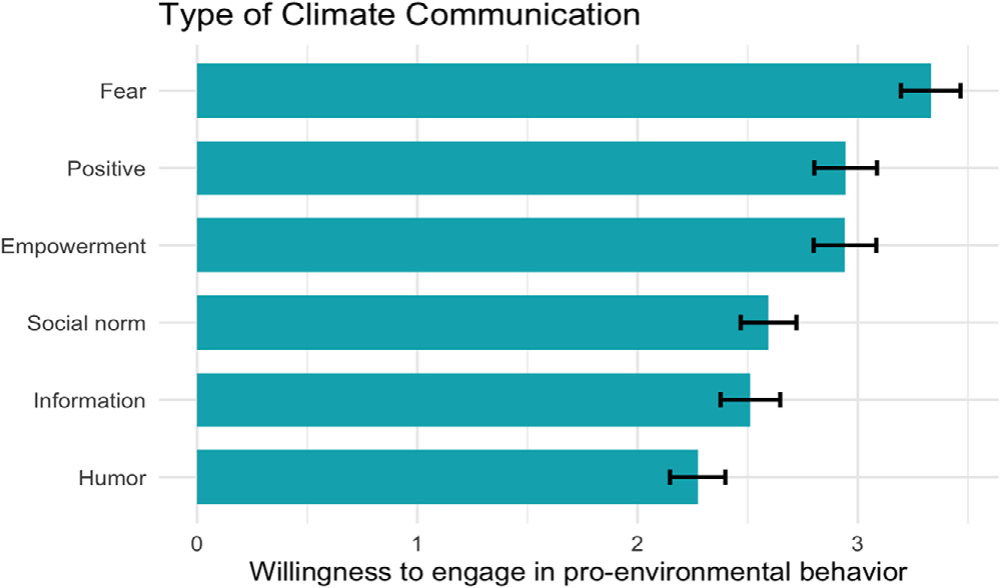
Willingness to engage in pro‐environmental behavior by communication strategy.

Bonferroni‐corrected pairwise comparisons of participants’ survey responses to climate communication strategies showed that youth reported different levels of willingness to engage in pro‐environmental behavior for different communication strategies (see Figure 2 and Table S1). Results indicate that fear‐based communication strategies elicited the highest willingness to engage in pro‐environmental behavior, followed by positive and empowering messages, while social norms, humor, and information were generally less effective. Differences between communication strategies were small to large (Cohen's *d*s = 0.19 to 0.77). These findings suggest that youth are more motivated by fear‐based, positive, and empowering messages over humor or factual information.

Our participatory focus group data indicate that youth have a need for reliable information about climate change in education and politics (Table 4, clusters 5–6). They wanted misinformation about climate change to be actively refuted. Moreover, youth described that they prefer climate change communication that is specific, refraining from vague statements such as “bad for the climate”; direct, explicating negative consequences of climate change for themselves; and positive: focusing on what is already going well and what still can be done in order to maintain positive future outlook. Youth emphasized that communication should avoid a directive tone and instead convey a sense of shared responsibility and togetherness.

#### 3.2.2 Youth Prefer Collective‐Systemic Over Individual Pro‐Environmental Behavior

We asked youth to indicate their preferences for pro‐environmental behaviors to address climate change (reanalyzed data from the Urban Rotterdam Project 2021 data; te Brinke & Crone, *manuscript under review*). A paired *t*‐test showed that collective‐systemic behavior scores (*M* = 1.68, *SD* = 1.17) were significantly higher than individual behavior scores (*M* = 0.75, *SD* = 0.94), *t*(1151) = 27.11, *p* < 0.001, 95% CI [0.86, 0.99], *M*diff = 0.92, Cohen's *d* = 0.79). This indicates that youth strongly prefer collective‐systemic behaviors (i.e. imposing rules for companies, producing more green energy, and creating more space for nature) over individual behaviors (i.e. eating fewer animal products, flying less, or buying fewer things).

Our participatory data furthermore indicate that youth preferred collective behavior because it gives them hope and motivation to discuss climate‐related worries with peers and to engage in pro‐environmental behavior together (Table 4, clusters 1, 2, and 4). Importantly, youth acknowledged the interconnectedness of individual and collective pro‐environmental behavior. That is, youth proposed interconnected climate action on multiple levels: individual (e.g. eating less meat) and collective (e.g. demanding sustainable company practices), supported by the government (e.g. reducing the price of vegetarian meat alternatives; Table 4, cluster 3). As one participant said: “As a society, we must work together on a shared vision: everyone participates, everyone contributes, generation after generation. We should not place the responsibility and concerns solely on us youth, because we often feel that we make little impact. Individual and collective responsibility also means raising the alarm with the government.”

In fact, youth stated that they wanted to be more involved in policymaking decisions (Table 4, cluster 7). For example, they proposed to embed youth participation in local and national policymaking, and to lower the voting age from 18 to 16 years. They encouraged the government to support youth organizations, to adequately reimburse youth representatives so that they can continue to exist, and to design a government‐led campaign to make contributing to societal challenges cool among youth. They also wished the government would more actively communicate with them what actions they already take to address climate change. Youth noted that collective or structural action can be motivated by using external “rewards” (e.g. cheaper vegetarian food, cheaper public transport; Table 4, cluster 6) that help individuals engage in more pro‐environmental behavior.

Youth emphasized the need for one structural change in particular: mandatory climate change education, embedded in the curriculum (Table 4, cluster 5). Youth wanted the government to ensure equal opportunities to learn about various climate‐related issues, such as the urgency and consequences of climate change across the globe; the impact of everyday behaviors on global warming; and how to make impactful sustainable personal and career decisions. Youth believed that climate change education could help them to hold themselves and others accountable and to engage in pro‐environmental behavior.

## 4 Discussion

Many adolescents and young adults worry about climate change (Hickman et al. 2021). A core challenge is helping youth live with that worry in ways that protect wellbeing and still support meaningful climate engagement. In this mixed method study, we combined survey data with youth participatory insights to examine how motivational quality shapes the worry to action link, and what kinds of climate communication and action pathways youth themselves prefer. Two patterns stand out. First, climate‐related worry was not uniformly linked to pro‐environmental behavior. Instead, the worry to behavior association depended on motivational quality, with stronger positive associations among youth who found pro‐environmental behavior interesting and enjoyable, or who felt internally pressured to act (e.g. out of guilt), while youth who acted because of external pressure showed no such link. Meanwhile, youth who had internalized pro‐environmental behavior as personally important showed a strong overall association with pro‐environmental behavior regardless of worry levels. Second, climate‐related worry was robustly associated with poorer affective wellbeing, and this association was not buffered by differences in pro‐environmental motivation. Finally, participants indicated to prefer climate communication that is direct and specific, and they indicated to prefer collective‐systemic mitigation pathways over individual pro‐environmental behaviors. Together, these findings illustrate how climate‐related worry can function as both burden and potential fuel for pro‐environmental behavior, and they point to intervention levers that align with youths’ need to contribute (Crone et al. 2024; Thomaes et al. 2023).

### 4.1 Worried Youth Engage in More Pro‐Environmental Behavior When Driven by Interest, Enjoyment or Internal Pressure

Consistent with recent work, the association between climate‐related worry and pro‐environmental behavior was conditioned by motivational quality (Qin et al. 2024; Sachisthal et al. 2025; Veijonaho et al. 2025; Wullenkord et al. 2023, 2025). Using self‐determination theory as a framework, our findings suggest that motivational quality helps explain when worry becomes constructive rather than disengaging (Deci and Ryan 2000; Ryan and Deci 2000, 2020; Spitzer et al. 2024).

Specifically, extrinsic motivation did not impact the association between climate‐related worry and pro‐environmental behavior, consistent with the hypothesis that external pressure and compliance are unlikely to sustain engagement. At the same time, we found that climate‐related worry was more strongly linked to pro‐environmental behavior among youth who reported to act out of interest or enjoyment (i.e. intrinsic motivation) and out of internal pressure (i.e. introjected motivation). Intrinsic motivation is a form of autonomous motivation that may help youth translate their climate‐related worry into pro‐environmental behavior, because they view such behavior as interesting, meaningful or enjoyable; worry may provide the additional urgency to act on that interest. Introjected motivation may also channel climate‐related worry into pro‐environmental behavior, as it operates through guilt and shame (Ryan and Deci 2020); affective processes that may be amplified by climate‐related worry. This interpretation fits with reports that youth experience morally tinged emotions such as guilt and shame around climate change, and that climate justice is central in youth climate movements (Hickman et al. 2021; Neas et al. 2022; van de Wetering & Lee, *manuscript under review*), potentially reflecting adolescents' growing concern with moral identity and social justice (Krettenauer and Victor 2017; Thomaes et al. 2023). Thus, both intrinsic and introjected motivation strengthened the worry‐behavior link, albeit through different motivational underpinnings: interest and enjoyment in the case of intrinsic motivation, and guilt and moral concern in the case of introjected motivation. However, future work should test whether introjected motivation—which is a form of controlled motivation—supports engagement at the cost of longer‐term wellbeing. This is particularly relevant given that climate‐related worry was robustly associated with poorer affective wellbeing in our data.

We also observed that youth who had internalized pro‐environmental behavior as personally important (identified motivation), another form of autonomous motivation, showed a strong direct association with pro‐environmental behavior, but attenuated the association with climate‐related worry. This does not mean that identified motivation is maladaptive. Rather, youth who have internalized pro‐environmental behavior as personally important engage regardless of their worry levels, leaving less room for worry to explain additional variance. This interpretation is compatible with the sustainability motive alignment hypothesis, which proposes that youth engage when pro‐environmental behavior aligns with their motives and personally endorsed goals (Grapsas et al. 2023; Thomaes et al. 2023; van de Wetering et al. 2025, 2025).

Together, these findings have implications for the design of interventions to promote youths’ pro‐environmental behavior. While existing interventions tend to focus on raising knowledge and awareness (Świątkowski et al. 2024; Van De Wetering et al. 2022), they may more effectively promote youth's pro‐environmental behavior and support their affective wellbeing when they aim to build intrinsic motivation. This can involve supporting autonomy through youth ownership over initiatives, supporting competence by making progress visible and recognizing youth contributions, and supporting relatedness by creating peer‐based opportunities for collective action (Spitzer et al. 2024; Thomaes et al. 2023; Świątkowski et al. 2024; Van De Wetering et al. 2022).

### 4.2 Youths’ Preferences for Climate Change Communication and Pro‐Environmental Action

Our participatory findings help interpret what youth consider motivating climate communication and action pathways. Across strategies, youth most strongly endorsed climate messages that combined (a) a clear articulation of risk and consequences (fear‐based content), with (b) pathways that highlight desirable outcomes (positive framing) and (c) actionable agency (empowerment). This pattern aligns with broader evidence that threat‐focused messages can motivate engagement when they are paired with effective and feasible response options, whereas threat without efficacy is more likely to evoke avoidance or defensive responses (Tannenbaum et al. 2015; Witte 1992; Witte and Allen 2000). Fear appeals can suppress engagement if they do not offer feasible response options (Nabi and Myrick 2019; O'Neill and Nicholson‐Cole 2009). One explanation for youth's preference for fear‐based communication is that youth in our study valued direct and specific communication. Such communication reduces psychological distance to climate change, which can increase personal relevance (Spence et al. 2012).

The high endorsement of empowerment and positive strategies suggests that youth desire agency and hope. Positive emotions such as hope are indeed associated with greater climate engagement, especially when hope is grounded in realistic pathways and shared agency rather than reassurance that “everything will be fine” (Geiger et al. 2023; Schneider et al. 2021). A practical implication is that positive framing may be most effective when coupled with empowerment, collective efficacy, and concrete near term actions.

The comparatively lower endorsement of information‐only communication is consistent with critiques that information is necessary but often insufficient for behavior change (Suldovsky 2017). Importantly, however, during focus groups, youth in our study still emphasized the need for reliable information, as well as skills and resources to actively refute misinformation, including misinformation encountered in political discourse. Youth's weaker endorsement of social norm messaging may reflect that descriptive norms can backfire if they imply that undesirable behavior is common. Finally, the lower endorsement for humor may indicate that humorous framing can risk trivializing a high stakes issue for some audiences, and that its effectiveness depends on format and audience characteristics. Experimental work shows that humor and satire can sometimes increase engagement or risk perceptions, but can also increase discounting or counterarguing depending on whether the humor is one sided or two sided and how it is interpreted (Anderson and Becker 2018; Skurka et al. 2022). Taken together, youth in our study preferred climate communication that is direct and specific, while also offering agency and collective pathways forward, rather than relying primarily on information, social norms, or humor.

Similar to adults, youth expressed a preference for collective over individual pro‐environmental behaviors (Nash et al. 2020). Youth in our study valued collective engagement with peers, because it felt more efficacious and supported their wellbeing, consistent with their developmental reorientation toward peers (Crone and Fuligni 2019). Nonetheless, youth acknowledged the interconnectedness of individual and collective pro‐environmental behavior, viewing individual behavior as meaningful when embedded in collective efforts and enabled by structural conditions (e.g. affordable meat alternatives; Atkinson and Jacquet 2021; Behrens 2025; Ridder and Thomaes 2025). Youth also voiced a desire to play an active role in collective and structural change, for example, through participatory mechanisms in local and national policymaking and by lowering the voting age to 16, also reflecting adolescents’ desire to matter and contribute meaningfully to society (Dahl et al. 2024; Fuligni 2019).

One implication is that inclusive sustainable transitions require policymakers to invite youth to the table and to communicate transparently about current and planned climate efforts. Youth want their perspectives to be heard, consistent with evidence that adolescents have a strong need to contribute and can be a powerful force in environmental change (Bandura and Cherry 2020; Fuligni 2019; te Brinke et al. 2025; Ridder and Thomaes 2025). Globally, many youth feel unsupported by their governments in the climate crisis, reporting that they do not take their concerns seriously enough (63.8%) and are failing young people (64.9%; Hickman et al. 2021). Consistent with our participatory findings, a nationally representative survey showed that a perceived lack of government action contributes to feelings of hopelessness and poses a growing barrier to pro‐environmental engagement among Dutch youth (Ipsos I&O 2025). Beyond participation, youth in our study emphasized concrete structural changes such as financial incentives for sustainable consumption (e.g. meat alternatives) and travel (e.g. public transport), as well as mandatory climate change education across all educational levels. Complementing recent recommendations (UNESCO 2022, 2024), they stressed the importance of education about local and global climate change consequences, and guidance on ways to contribute, including how to recognize and counter misinformation.

### 4.3 Strengths, Limitations, and Future Directions

By adopting a mixed method design, the present study integrates theory‐driven analyses with youths’ lived experiences and perspectives. Using a self‐determination theory lens, the survey component provides a needs‐based account of the motivational conditions under which climate‐related worry is more likely to co‐occur with pro‐environmental behavior across youth of diverse ages, genders, and educational and cultural backgrounds. Complementary, the youth participatory component clarifies what youth themselves consider motivating and feasible, including preferences for direct and specific communication, reliable information and misinformation literacy, and collective and systemic pathways to climate action. Documenting youths’ experiences and solutions can enrich interpretation of quantitative patterns and help ensure that scientific and policy recommendations remain aligned with youths’ needs and realities (Toenders et al. 2024; UNDP 2021).

Our study also has limitations. First, the survey data in this study were cross‐sectional, which prevents causal or time‐change related conclusions. While we theorize that climate‐related worry predicts increased pro‐environmental behavior, the reverse may also be true: youth who engage in more pro‐environmental behavior may experience more climate‐related worry over time (Teo et al. 2024; Veijonaho et al. 2025). Future research may employ longitudinal designs to unravel within‐person developments of climate‐related worry and pro‐environmental behavior. Such research could also examine how needs‐aligned (or: motive‐aligned) interventions may promote youth's pro‐environmental behavior and support their wellbeing over time, potentially via increased need satisfaction and, subsequently, self‐determined motivation (Spitzer et al. 2024; Thomaes et al. 2023).

Second, although the study measures were designed to be accessible across a broad age range (12–30 years, although the majority of participants were aged between 16 and 22), some items may have been more cognitively demanding for younger participants. While clarifying instructions were provided and scale reliabilities were satisfactory, future research could strengthen both developmental appropriateness and construct validity by more explicitly assessing young participants’ understanding of items, for example, through co‐interpretation of questionnaire items. In addition, our finding that youth in our study preferred fear‐related climate messaging may partly reflect a measurement artifact, as projecting consequences 100 years into the future may not have elicited high levels of fear due to temporal discounting of distant climate impacts (Berry et al. 2017).

Third, due to practical constraints within a larger survey project, pro‐environmental behavior was assessed using a single self‐report item assessing youths’ perception of how often they do things that benefit the climate. While prior validation work indicates that single‐item measures can show acceptable validity in survey research for broad constructs (Robins et al. 2001; Wanous et al. 1997), future research should replicate these findings using multi‐item measures that distinguish between different forms of pro‐environmental behavior (e.g. individual and collective) or incorporate more objective behavioral indicators (e.g. observations; Lange and Dewitte 2019). In addition, wellbeing was operationalized as affect or general mood, whereas wellbeing is multidimensional, encompassing hedonic and eudaimonic aspects as well as cognitive and affective components (Bautista et al. 2023; Green et al. 2023). Future research should test whether the observed associations generalize to other wellbeing facets, such as life satisfaction, meaning, or psychological functioning.

Fourth, while it is a strength of the present study that participants were sampled from diverse sociocultural backgrounds, our data were limited to youth in the Netherlands and may reflect some self‐selection, as participants were recruited via schools, community centers, and youth organizations and knew the sessions would focus on climate related topics. To further explore the heterogeneity in the relation between climate worry and wellbeing in adolescents, future research should consider additional social and contextual factors, such as experiences with climate disasters in the past. Future studies should also continue efforts to include youth from different countries, especially from the Global South, given that (youth's perceptions of) climate change impacts as well as the adoption of climate‐related educational interventions vary between countries (Hickman et al. 2021; Thiery et al. 2021; UNESCO 2021). At the same time, our findings held when controlling for youth's age, educational level, or cultural background within our sample. This is consistent with the idea that climate change is a pervasive stressor for youth, cutting across demographic categories (Coffey et al. 2021; Hickman et al. 2021; Pihkala 2020), and that variation in engagement is better explained by motivational processes (De Groot and Steg 2010; Bamberg and Möser 2007).

Given youth's preference for collective‐systemic pro‐environmental behavior in our study, we recommend future research to prioritize the development of interventions that promote motivation for collective pro‐environmental behavior, such as participating in climate protests, sharing perspectives with policymakers, and discussing concerns with peers, rather than focusing primarily on individual behavior change, as most current interventions do (Van De Wetering et al. 2022). Aligning with youth's expressed need for support, such research may also investigate interventions or strategies to motivate and enable authorities and other adults to support youth in collective pro‐environmental efforts. From a developmental systems perspective, youth require supportive social networks, institutions, and access to resources to support their societal engagement (Pereira and Freire 2021; Schill et al. 2019).

## 5 Conclusion

Young people are increasingly viewed as agents of change in the climate crisis, yet many carry the weight of worry about it. The present study combined survey and participatory methods to examine how motivational quality shapes the links between climate‐related worry, pro‐environmental behavior, and affective wellbeing, and to document youths' preferences for climate communication and action pathways. We found that climate‐related worry was more strongly associated with pro‐environmental behavior among youth motivated by interest and enjoyment or by internal pressure such as guilt, although whether such internal pressure supports sustainable engagement or comes at a wellbeing cost remains to be tested. Youth who had internalized pro‐environmental behavior as personally important engaged in pro‐environmental behavior regardless of their worry levels. At the same time, climate‐related worry was robustly associated with reduced affective wellbeing across all motivation types. Participatory data further revealed that youth prefer direct, risk‐emphasizing, positive, and empowering climate communication, as well as collective‐systemic over individual pro‐environmental behaviors. Overall, our research illustrates the importance of supporting youths' motivation to help them turn climate‐related worry into pro‐environmental behavior, while also presenting youths' own perspectives on what motivating climate communication and action might look like, and underscoring the value of involving youth as active contributors to research and evidence‐informed climate action.

## Author Contributions

**Anne–Wil Kramer**: investigation, writing – original draft, methodology, visualization, conceptualization, formal analysis, project administration, data curation. **Judith van de Wetering**: writing – original draft, visualization, writing – review and editing. **Suzanne van de Groep**: conceptualization, investigation, writing – review and editing, methodology. **Lysanne W. te Brinke**: conceptualization, investigation, methodology, writing – review and editing. **Yara J. Toenders**: writing – review and editing, methodology, conceptualization. **Kayla H. Green**: methodology, writing – review and editing, conceptualization. **Eveline A. Crone**: conceptualization, funding acquisition, writing – review and editing, methodology, supervision.

## Funding

This project is funded by the Dutch Research Council (NWA.1397.21221.003) and by the Muller Foundation, a named fund at the Erasmus Trustfonds.

## Ethics Statement

This study was conducted in accordance with the declaration of Helsinki. The studies in this manuscript were approved by the institutional ethical review board of the Erasmus University Rotterdam, the Netherlands.

## Conflicts of Interest

The authors declare no conflicts of interest.

## Supporting information




**Supporting File 1**: desc70217‐sup‐0001‐SuppMat.docx.

## Data Availability

The data are not publicly available due to privacy or ethical restrictions.

## References

[desc70217-bib-0002] Anderson, A. A. , and A. B. Becker . 2018. “Not Just Funny After All: Sarcasm as a Catalyst for Public Engagement With Climate Change.” Science Communication 40, no. 4: 524–540.

[desc70217-bib-0001] Anyon, Y. , K. Bender , H. Kennedy , and J. Dechants . 2018. “A Systematic Review of Youth Participatory Action Research (YPAR) in the United States: Methodologies, Youth Outcomes, and Future Directions.” Health Education & Behavior 45, no. 6: 865–878. 10.1177/1090198118769357. 29749267

[desc70217-bib-0004] Ardoin, N. M. , A. W. Bowers , N. W. Roth , and N. Holthuis . 2018. “Environmental Education and K‐12 Student Outcomes: A Review and Analysis of Research.” Journal of Environmental Education 49, no. 1: 1–17.

[desc70217-bib-0003] Atkinson, Q. D. , and J. Jacquet . 2021. “Challenging the Idea That Humans Are Not Designed to Solve Climate Change.” Perspectives on Psychological Science 17, no. 3: 619–630. 10.1177/17456916211018454. 34738846

[desc70217-bib-0006] Bamberg, S. , and G. Möser . 2007. “Twenty Years After Hines, Hungerford, and Tomera: A New Meta‐Analysis of Psycho‐Social Determinants of Pro‐Environmental Behaviour.” Journal of Environmental Psychology 27, no. 1: 14–25.

[desc70217-bib-0005] Bandura, A. , and L. Cherry . 2020. “Enlisting the Power of Youth for Climate Change.” American Psychologist 75, no. 7: 945–951. 10.1037/amp0000512. 31436438

[desc70217-bib-0007] Bautista, T. G. , G. Roman , M. Khan , et al. 2023. “What Is Well‐being? A Scoping Review of the Conceptual and Operational Definitions of Occupational Well‐Being.” Journal of Clinical and Translational Science 7, no. 1: e227. 38028344 10.1017/cts.2023.648 PMC10643923

[desc70217-bib-0008] Becht, A. , J. Spitzer , S. Grapsas , et al. 2024. “Feeling Anxious and Being Engaged in a Warming World: Climate Anxiety and Adolescents' Pro‐Environmental Behavior.” Journal of Child Psychology and Psychiatry, ahead of print. 10.1111/jcpp.14035. 38940197

[desc70217-bib-0009] Behrens, S. 2025. ““It's Like Getting Someone Who's Been Hit by a Car to Run a Speed Awareness Course”: How Young Activists in the UK Make Sense of Personal, Collective and Institutional Agency.” Youth & Society ahead of print, 0044118X251346801. 10.1177/0044118X251346801.

[desc70217-bib-0010] Berry, M. S. , N. P. Nickerson , and A. L. Odum . 2017. “Delay Discounting as an Index of Sustainable Behavior: Devaluation of Future Air Quality and Implications for Public Health.” International Journal of Environmental Research and Public Health 14, no. 9: 997. 10.3390/ijerph14090997. 28862671 PMC5615534

[desc70217-bib-0011] Boykoff, M. , and B. Osnes . 2019. “A Laughing Matter? Confronting Climate Change Through Humor.” Political Geography 68: 154–163.

[desc70217-bib-0117] Braun, V. , and V. Clarke . 2019. “Reflecting on Reflexive Thematic Analysis.” Qualitative Research in Sport, Exercise And Health 11, no. 4: 589–597.

[desc70217-bib-0012] Brinke, L. W. t. , S. W. Sweijen , and E. A. Crone . 2025. “Harnessing Youths' Need to Contribute to Societal Challenges: A Naturalistic Experiment.” Journal of Adolescence 97: 1547–1556. https://doi‐org.eur.idm.oclc.org/10.1002/jad.12517. 40484978 10.1002/jad.12517 PMC12318459

[desc70217-bib-0013] Braun, V. , and V. Clarke . 2006. “Using Thematic Analysis in Psychology.” Qualitative Research in Psychology 3, no. 2: 77–101.

[desc70217-bib-0015] Coffey, Y. , N. Bhullar , J. Durkin , M. S. Islam , and K. Usher . 2021. “Understanding Eco‐Anxiety: A Systematic Scoping Review of Current Literature and Identified Knowledge Gaps.” Journal of Climate Change and Health 3: 100047.

[desc70217-bib-0016] Crone, E. A. , and A. J. Fuligni . 2019. “Self and Others in Adolescence.” Annual Review of Psychology 71, no. 1: 447–469. 10.1146/annurev-psych-010419. 31337274

[desc70217-bib-0017] Crone, E. A. , S. v. d. Groep , and L. W. t. Brinke . 2024. “Can Adolescents be Game Changers for 21st‐Century Societal Challenges?” Trends in Cognitive Sciences 28, no. 6: 484–486. 10.1016/j.tics.2024.04.006. 38744600

[desc70217-bib-0018] Dahl, R. E. , E. Armstrong‐Carter , and W. van den Bos . 2024. “Wanting to Matter and Learning to Care: A Neurodevelopmental Window of Opportunity for (pro)Social Learning?” Developmental Cognitive Neuroscience 69: 101430. 10.1016/j.dcn.2024.101430. 39151254 PMC11377138

[desc70217-bib-0019] De Groot, J. I. , and L. Steg . 2010. “Relationships Between Value Orientations, Self‐Determined Motivational Types and Pro‐Environmental Behavioural Intentions.” Journal of Environmental Psychology 30, no. 4: 368–378.

[desc70217-bib-0020] Deci, E. L. , and R. M. Ryan . 2000. “The" What" and" Why" of Goal Pursuits: Human Needs and the Self‐Determination of Behavior.” Psychological Inquiry 11, no. 4: 227–268.

[desc70217-bib-0021] de Moor, J. , K. Uba , M. Wahlström , M. Wennerhag , and M. D. Vydt . 2020. Protest for a Future II: Composition, Mobilization and Motives of the Participants in Fridays For Future Climate Protests on 20‐27 September, 2019, in 19 Cities Around the World. https://www.diva‐portal.org/smash/record.jsf?pid=diva2%3A1397070&dswid=‐9325.

[desc70217-bib-0022] Donati, M. A. , S. Santisi , L. Di Leonardo , and C. Primi . 2025. “How to Measure Climate Change Worry in Adolescents? Psychometric Properties of the Climate Change Worry Scale.” International Journal of Behavioral Development 49, no. 2: 195–203. 10.1177/01650254241266119.

[desc70217-bib-0024] Fuligni, A. J. 2019. “The Need to Contribute During Adolescence.” Perspectives on Psychological Science 14, no. 3: 331–343. 10.1177/1745691618805437. 30562473 PMC6497551

[desc70217-bib-0118] Geiger, N. , T. Dwyer , and J. K. Swim . 2023. “Hopium or Empowering Hope? A Meta‐Analysis of Hope and Climate Engagement.” Frontiers in Psychology 14: 1139427. 37649687 10.3389/fpsyg.2023.1139427 PMC10465179

[desc70217-bib-0026] Grapsas, S. , J.v.d. Wetering , J. Spitzer , A. M. G. Poorthuis , and S. Thomaes . 2023. “When Sustainability Aligns With Adolescent Motives: Development and Validation of the Sustainability Motive‐Alignment Scale (SMAS).” Applied Developmental Science 28, no. 4: 612–633. 10.1080/10888691.2023.2260748.

[desc70217-bib-0027] Green, K. H. , A. I. Becht , S. van de Groep , R. van der Cruijsen , S. W. Sweijen , and E. A. Crone . 2023. “Socioeconomic Hardship, Uncertainty About the Future, and Adolescent Mental Wellbeing Over a Year During the COVID‐19 Pandemic.” Social Development 32, no. 3: 1092–1114. 10.1111/sode.12674.

[desc70217-bib-0028] Green, K. H. , S. van de Groep , R. van der Cruijsen , M. G. Polak , and E. A. Crone . 2023. “The Multidimensional Wellbeing in Youth Scale (MWYS): Development and Psychometric Properties.” Personality and Individual Differences 204: 112038.

[desc70217-bib-0029] Hampton, S. , and L. Whitmarsh . 2023. “Choices for Climate Action: A Review of the Multiple Roles Individuals Play.” One Earth 6, no. 9: 1157–1172.

[desc70217-bib-0030] Hickman, C. , E. Marks , P. Pihkala , et al. 2021. “Climate Anxiety in Children and Young People and Their Beliefs About Government Responses to Climate Change: A Global Survey.” Lancet Planetary Health 5: e863–e873. 10.1016/S2542-5196(21)00278-3. 34895496

[desc70217-bib-0031] Holtzblatt, K. , and H. Beyer . 2017. Contextual Design: Design for Life. 2nd ed. Morgan Kaufmann.

[desc70217-bib-0032] Ipsos I&O . 2025. Duurzaam denken, duurzaam doen. https://206.wpcdnnode.com/ipsos‐publiek.nl/wp‐content/uploads/2025/06/rapport‐duurzaam‐denken‐duurzaam‐doen‐2025‐onder‐embargo.pdf.

[desc70217-bib-0033] Kramer, A. W. , H. M. Huizenga , A. C. Van Duijvenvoorde , and L. Krabbendam . 2024. “Do I Want to Learn Today? Day‐to‐Day Variations in Adolescents' Academic Motivation and Effort.” Learning and Motivation 85: 101957.

[desc70217-bib-0034] Kramer, A. W. , J. V. Schaaf , and H. M. Huizenga . 2023. “How Much Do You Want to Learn? High‐School Students' Willingness to Invest Effort in Valenced Feedback‐Learning Tasks.” Learning and Individual Differences 108: 102375.

[desc70217-bib-0035] Kramer, A. W. , A. C. Van Duijvenvoorde , L. Krabbendam , and H. M. Huizenga . 2021. “Individual Differences in Adolescents' Willingness to Invest Cognitive Effort: Relation to Need for Cognition, Motivation and Cognitive Capacity.” Cognitive Development 57: 100978.

[desc70217-bib-0036] Kramer, A. W. , L. Krabbendam , J. V. Schaaf , H. M. Huizenga , and A. C. Van Duijvenvoorde . 2025. “Make It Worth It: Effort‐Reward Modulations on Reinforcement‐Learning and Prediction‐Error Signaling Across Adolescence.” Developmental Cognitive Neuroscience 73: 101559. 40306168 10.1016/j.dcn.2025.101559 PMC12063155

[desc70217-bib-0037] Krettenauer, T. , and R. Victor . 2017. “Why be Moral? Moral Identity Motivation and Age.” Developmental Psychology 53, no. 8: 1589–1596. 10.1037/dev0000353. 28517946

[desc70217-bib-0114] Krettenauer, T. , W. Wang , F. Jia , and Y. Yao . 2020. “Connectedness With Nature and the Decline of Pro‐Environmental Behavior in Adolescence: A Comparison of Canada and China.” Journal of Environmental Psychology 71: 101348.

[desc70217-bib-0038] Lange, F. , and S. Dewitte . 2019. “Measuring Pro‐Environmental Behavior: Review and Recommendations.” Journal of Environmental Psychology 63: 92–100.

[desc70217-bib-0039] Lee, K. , N. Gjersoe , S. O'Neill , and J. Barnett . 2020. “Youth Perceptions of Climate Change: A Narrative Synthesis.” Wiley Interdisciplinary Reviews: Climate Change 11, no. 3: e641. 10.1002/wcc.641.

[desc70217-bib-0040] Levitt, H. M. , M. Bamberg , J. W. Creswell , D. M. Frost , R. Josselson , and C. Suárez‐Orozco . 2018. “Journal Article Reporting Standards for Qualitative Primary, Qualitative Meta‐Analytic, and Mixed Methods Research in Psychology: The APA Publications and Communications Board Task Force Report.” American Psychologist 73, no. 1: 26. 29345485 10.1037/amp0000151

[desc70217-bib-0041] Martela, F. , and K. M. Sheldon . 2019. “Clarifying the Concept of Well‐Being: Psychological Need Satisfaction as the Common Core Connecting Eudaimonic and Subjective Well‐Being.” Review of General Psychology 23, no. 4: 458–474. 10.1177/1088868319860276.

[desc70217-bib-0042] Milyavskaya, M. , M. Inzlicht , N. Hope , and R. Koestner . 2015. “Saying “no” to Temptation: Want‐to Motivation Improves Self‐Regulation by Reducing Temptation Rather Than by Increasing Self‐Control.” Journal of Personality and Social Psychology 109, no. 4: 677. 25984785 10.1037/pspp0000045

[desc70217-bib-0043] Nabi, R. L. , and J. G. Myrick . 2019. “Uplifting Fear Appeals: Considering the Role of Hope in Fear‐Based Persuasive Messages.” Health Communication 34, no. 4: 463–474. 29313717 10.1080/10410236.2017.1422847

[desc70217-bib-0044] Nash, N. , L. Whitmarsh , S. Capstick , et al. 2020. “Local Climate Change Cultures: Climate‐Relevant Discursive Practices in Three Emerging Economies.” Climatic Change 163, no. 1: 63–82. 33281250 10.1007/s10584-019-02477-8 PMC7704444

[desc70217-bib-0045] Neas, S. , A. Ward , and B. Bowman . 2022. “Young People's Climate Activism: A Review of the Literature.” Frontiers in Political Science 4: 940876. 10.3389/fpos.2022.940876.

[desc70217-bib-0046] Ng, J. Y. , N. Ntoumanis , C. Thøgersen‐Ntoumani , et al. 2012. “Self‐Determination Theory Applied to Health Contexts: A Meta‐Analysis.” Perspectives on Psychological Science 7, no. 4: 325–340. 26168470 10.1177/1745691612447309

[desc70217-bib-0047] Ogunbode, C. A. , R. Doran , D. Hanss , T. Kauppinen , L.‐O. Johansson , and K. Salmela‐Aro . 2022. “Climate Anxiety, Pro‐Environmental Action and Wellbeing: An International Study.” Journal of Environmental Psychology 81: 101784. 10.1016/j.jenvp.2022.101784.

[desc70217-bib-0048] Ogunbode, C. A. , R. Doran , D. Hanss , et al. 2022. “Climate Anxiety, Wellbeing and Pro‐Environmental Action: Correlates of Negative Emotional Responses to Climate Change in 32 Countries.” Journal of Environmental Psychology 84: 101887. 10.1016/J.JENVP.2022.101887.

[desc70217-bib-0049] Ojala, M. 2022. “Hope and Climate‐Change Engagement From a Psychological Perspective.” Current Opinion in Psychology 49: 101514. 10.1016/j.copsyc.2022.101514. 36502586

[desc70217-bib-0050] Ojala, M. 2023. “How Do Children, Adolescents, and Young Adults Relate to Climate Change? Implications for Developmental Psychology.” European Journal of Developmental Psychology 20, no. 6: 929–943. 10.1080/17405629.2022.2108396.

[desc70217-bib-0051] Olsson, D. , N. Gericke , and S. N. Chang Rundgren . 2016. “The Effect of Implementation of Education for Sustainable Development in Swedish Compulsory Schools–Assessing Pupils' Sustainability Consciousness.” Environmental Education Research 22, no. 2: 176–202.

[desc70217-bib-0052] O'neill, S. , and S. Nicholson‐Cole . 2009. ““Fear Won't Do It” Promoting Positive Engagement With Climate Change Through Visual and Iconic Representations.” Science Communication 30, no. 3: 355–379.

[desc70217-bib-0053] Ozer, E. J. , M. Abraczinskas , A. B. Suleiman , H. Kennedy , and A. Nash . 2024. “Youth‐Led Participatory Action Research and Developmental Science: Intersections and Innovations.” Annual Review of Developmental Psychology 6: 401–423. 10.1146/annurev-devpsych-010923-100158.

[desc70217-bib-0054] Pelletier, L. G. , K. M. Tuson , I. Green‐Demers , K. Noels , and A. M. Beaton . 1998. “Why Are You Doing Things for the Environment? The Motivation Toward the Environment Scale (MTES).” Journal of Applied Social Psychology 28, no. 5: 437–468. 10.1111/j.1559-1816.1998.tb01714.x.

[desc70217-bib-0055] Pereira, T. , and T. Freire . 2021. “Positive Youth Development in the Context of Climate Change: A Systematic Review.” Frontiers in Psychology 12: 786119. 10.3389/fpsyg.2021.786119. 34887822 PMC8649636

[desc70217-bib-0056] Pihkala, P. 2020. “Eco‐anxiety and Environmental Education.” Sustainability 12, no. 23: 10149.

[desc70217-bib-0057] Poortinga, W. , L. Whitmarsh , L. Steg , G. Böhm , and S. Fisher . 2019. “Climate Change Perceptions and Their Individual‐Level Determinants: A Cross‐European Analysis.” Global Environmental Change 55: 25–35. 10.1016/j.gloenvcha.2019.01.007.

[desc70217-bib-0058] Qin, Z. , Q. Wu , C. Bi , Y. Deng , and Q. Hu . 2024. “The Relationship Between Climate Change Anxiety and Pro‐Environmental Behaviors in Adolescents: The Mediating Role of Future Self‐Continuity and the Moderating Role of Green Self‐Efficacy.” BMC Psychology 12: 241. 10.1186/s40359-024-01746-1. 38678287 PMC11056057

[desc70217-bib-0059] Ramadan, R. , A. Randell , S. Lavoie , et al. 2023. “Empirical Evidence for Climate Concerns, Negative Emotions and Climate‐related Mental Ill‐Health in Young People: A Scoping Review.” Early Intervention in Psychiatry 17, no. 6: 537–563. 10.1111/eip.13374. 36641809

[desc70217-bib-0060] R Core Team . 2024. R: A Language and Environment for Statistical Computing (Version 4.5.1) [Computer software]. R Foundation for Statistical Computing. https://www.R‐project.org/.

[desc70217-bib-0061] Renaud‐Dubé, A. , G. Taylor , N. Lekes , R. Koestner , and F. Guay . 2010. “Adolescents' Motivation Toward the Environment: Age‐Related Trends and Correlates.” Canadian Journal of Behavioural Science 42, no. 3: 194.

[desc70217-bib-0062] Ridder, D.d. , and S. Thomaes . 2025. “When and How Behavior Change Can Accelerate System Change (and Vice Versa): Mapping Reciprocal Processes for Climate Change Mitigation.” Behavioural Public Policy 1007: 1–18. 10.1017/bpp.2025.10007.

[desc70217-bib-0063] Robins, R. W. , H. M. Hendin , and K. H. Trzesniewski . 2001. “Measuring Global Self‐Esteem: Construct Validation of a Single‐Item Measure and the Rosenberg Self‐Esteem Scale.” Personality and Social Psychology Bulletin 27, no. 2: 151–161.

[desc70217-bib-0065] Ryan, R. M. , and E. L. Deci . 2000. “Self‐Determination Theory and the Facilitation of Intrinsic Motivation, Social Development, and Well‐Being.” American Psychologist 55, no. 1: 68–78. 10.1037/0003-066X.55.1.68. 11392867

[desc70217-bib-0066] Ryan, R. M. , and E. L. Deci . 2020. “Intrinsic and Extrinsic Motivation From a Self‐Determination Theory Perspective: Definitions, Theory, Practices, and Future Directions.” Contemporary Educational Psychology 61: 101860.

[desc70217-bib-0115] Ryan, R. M. , J. J. Duineveld , S. I. Di Domenico , W. S. Ryan , B. A. Steward , and E. L. Bradshaw . 2022. “We Know This Much is (Meta‐Analytically) True: A Meta‐Review of Meta‐Analytic Findings Evaluating Self‐Determination Theory.” Psychological Bulletin 148, no. 11–12: 813.

[desc70217-bib-0067] Sachisthal, M. S. M. , J. N. Zadelaar , and M. E. J. Raijmakers . 2025. “A Psychological Network Approach to Engagement With Climate Change in Dutch Youth.” Acta Psychologica 258: 105290. 10.1016/j.actpsy.2025.105290. 40680700

[desc70217-bib-0068] Sandelowski, M. 2001. “Real Qualitative Researchers Do Not Count: The Use of Numbers in Qualitative Research.” Research in Nursing & Health 24, no. 3: 230–240. 11526621 10.1002/nur.1025

[desc70217-bib-0069] Sanson, A. V. , J. V. Hoorn , and S. E. L. Burke . 2019. “Responding to the Impacts of the Climate Crisis on Children and Youth.” Child Development Perspectives 13, no. 4: 201–207. 10.1111/cdep.12342.

[desc70217-bib-0070] Sanson, A. , and M. Bellemo . 2021. “Children and Youth in the Climate Crisis.” British Journal of Psychiatry Bulletin 45, no. 4: 205–209. 10.1192/bjb.2021.16. PMC8499628 33879278

[desc70217-bib-0071] Sawyer, S. M. , P. S. Azzopardi , D. Wickremarathne , and G. C. Patton . 2018. “The Age of Adolescence.” Lancet Child and Adolescent Health 2, no. 3: 223–228. 10.1016/S2352-4642(18)30022-1. 30169257

[desc70217-bib-0072] Schill, C. , J. M. Anderies , T. Lindahl , et al. 2019. “A More Dynamic Understanding of Human Behaviour for the Anthropocene.” Nature Sustainability 2, no. 12: 1075–1082. 10.1038/s41893-019-0419-7.

[desc70217-bib-0073] Schneider, C. R. , L. Zaval , and E. M. Markowitz . 2021. “Positive Emotions and Climate Change.” Current Opinion in Behavioral Sciences 42: 114–120.

[desc70217-bib-0074] Schunk, D. H. , P. R. Pintrich , and J. L. Meece . 2014. Motivation in Education: Theory, Research, and Applications. Pearson.

[desc70217-bib-0075] Solmi, M. , J. Radua , M. Olivola , et al. 2022. “Age at Onset of Mental Disorders Worldwide: Large‐Scale Meta‐Analysis of 192 Epidemiological Studies.” Molecular Psychiatry 27, no. 1: 281–295. 10.1038/s41380-021-01161-7. 34079068 PMC8960395

[desc70217-bib-0076] Skurka, C. , R. Romero‐Canyas , H. H. Joo , D. Acup , and J. Niederdeppe . 2022. “Emotional Appeals, Climate Change, and Young Adults: A Direct Replication of Skurka et al. (2018).” Human Communication Research 48, no. 1: 147–156.

[desc70217-bib-0077] Spence, A. , W. Poortinga , and N. Pidgeon . 2012. “The Psychological Distance of Climate Change.” Risk Analysis: An International Journal 32, no. 6: 957–972. 10.1111/j.1539-6924.2011.01695.x 21992607

[desc70217-bib-0078] Spitzer, J. , S. Grapsas , A. M. G. Poorthuis , and S. Thomaes . 2023. “Supporting Youth Emotionally When Communicating About Climate Change: A Self‐Determination Theory Approach.” International Journal of Behavioral Development 48, no. 2: 113–124. 10.1177/01650254231190919.

[desc70217-bib-0079] Spitzer, J. , S. Grapsas , A. M. G. Poorthuis , M. Vansteenkiste , and S. Thomaes . 2024. “Coming of Age in a Warming World: A Self‐Determination Theory Perspective.” Child Development Perspectives 48, no. 2. 10.1111/CDEP.12534.

[desc70217-bib-0082] Stern, P. C. 2000. “New Environmental Theories: Toward a Coherent Theory of Environmentally Significant Behavior.” Journal of Social Issues 56, no. 3: 407–424.

[desc70217-bib-0080] Stewart, A. E. 2021. “Psychometric Properties of the Climate Change Worry Scale.” International Journal of Environmental Research and Public Health 18, no. 2: 494. 33435348 10.3390/ijerph18020494 PMC7826965

[desc70217-bib-0081] Suldovsky, B. 2017. “The Information Deficit Model and Climate Change communication.” In Oxford Research Encyclopedia of Climate Science, edited by S. R. Wilson . Oxford University Press.

[desc70217-bib-0083] Świątkowski, W. , F. L. Surret , J. Henry , C. Buchs , E. P. Visintin , and F. Butera . 2024. “Interventions Promoting Pro‐environmental Behaviors in Children: A Meta‐Analysis and a Research Agenda.” Journal of Environmental Psychology 96: 102295.

[desc70217-bib-0084] Tannenbaum, M. B. , J. Hepler , R. S. Zimmerman , et al. 2015. “Appealing to Fear: A Meta‐Analysis of Fear Appeal Effectiveness and Theories.” Psychological Bulletin 141, no. 6: 1178. 26501228 10.1037/a0039729 PMC5789790

[desc70217-bib-0085] Teo, S. M. , C. X. Gao , N. Brennan , et al. 2024. “Climate Change Concerns Impact on Young Australians' Psychological Distress and Outlook for the Future.” Journal of Environmental Psychology 93: 102209. 10.1016/j.jenvp.2023.102209.

[desc70217-bib-0086] Terry, G. , N. Hayfield , V. Clarke , and V. Braun . 2017. “Thematic Analysis.” SAGE Handbook of Qualitative Research in Psychology 2, no. 17‐37: 25.

[desc70217-bib-0087] Thiery, W. , S. Lange , J. Rogelj , et al. 2021. “Intergenerational Inequities in Exposure to Climate Extremes.” Science 374, no. 6564: 158–160. 10.1126/science.abi7339. 34565177

[desc70217-bib-0088] Thomaes, S. , S. Grapsas , J.v.d. Wetering , J. Spitzer , and A. Poorthuis . 2023. “Green Teens: Understanding and Promoting Adolescents' Sustainable Engagement.” One Earth 6, no. 4: 352–361. 10.1016/j.oneear.2023.02.006.

[desc70217-bib-0113] Thomaes, S. 2026. “Climate Change and Youth Development: A View of an Emerging Field.” International Journal of Behavioral Development 50, no. 2: 264–271. 41767709 10.1177/01650254251317141 PMC12948032

[desc70217-bib-0089] Toenders, Y. J. , K. H. Green , L. W. t. Brinke , R. v. d. Cruijsen , S. v. d. Groep , and E. A. Crone . 2024. “From Developmental Neuroscience to Policy: A Novel Framework Based on Participatory Research.” Developmental Cognitive Neuroscience 101398. 10.1016/J.DCN.2024.101398. 38850964 PMC11200278

[desc70217-bib-0090] UNDP . 2021. *The Peoples’ Climate Vote*. UNDP. https://www.undp.org/content/undp/en/home/librarypage/climate‐and‐disaster‐resilience‐/The‐Peoples‐Climate‐Vote‐Results.html.

[desc70217-bib-0091] UNESCO . 2022. Youth Demands for Quality Climate Change Education . https://unesdoc.unesco.org/ark:/48223/pf0000383615.

[desc70217-bib-0092] UNESCO . 2024. Greening Curriculum Guidance–Teaching and Learning for Climate Action . 10.54675/aooz1758.

[desc70217-bib-0093] UNICEF . 2021. The Climate Crisis Is a Child Rights Crisis: Introducing the Children's Climate Risk Index .

[desc70217-bib-0094] UNESCO . 2021. *Getting Every School Climate‐Ready: How Countries Are Integrating Climate Change Issues in Education* (ED‐2021/WS/35). 10.54675/NBHC8523.

[desc70217-bib-0095] United Nations . 1989. Convention on the Rights of the Child . https://www.ohchr.org/en/instruments‐mechanisms/instruments/convention‐rights‐child.

[desc70217-bib-0096] Vansteenkiste, M. , E. Sierens , B. Soenens , K. Luyckx , and W. Lens . 2009. “Motivational Profiles From a Self‐Determination Perspective: The Quality of Motivation Matters.” Journal of Educational Psychology 101, no. 3: 671.

[desc70217-bib-0097] Van De Wetering, J. , P. Leijten , J. Spitzer , and S. Thomaes . 2022. “Does Environmental Education Benefit Environmental Outcomes in Children and Adolescents? A Meta‐Analysis.” Journal of Environmental Psychology 81: 101782.

[desc70217-bib-0098] van de Wetering, J. , S. Grapsas , A. Poorthuis , and S. Thomaes . 2025. “Shifting Peer Norms, Shifting Behavior? Optimizing Educational Interventions to Promote Adolescents' Sustainable Dietary Choices.” Journal of Environmental Psychology 109: 102880.

[desc70217-bib-0116] van de Wetering, J. , S. Grapsas , A. Poorthuis , and S. Thomaes . 2025. “Promoting Adolescents' Pro‐Environmental Behavior: A Motive‐Alignment Approach.” Journal of Research on Adolescence 35, no. 1: e13044. 39658356 10.1111/jora.13044 PMC11758481

[desc70217-bib-0100] van der Werff, E. , L. Steg , and K. Keizer . 2013. “The Value of Environmental Self‐Identity: The Relationship Between Biospheric Values, Environmental Self‐Identity and Environmental Preferences, Intentions and Behaviour.” Journal of Environmental Psychology 34: 55–63. 10.1016/j.jenvp.2012.12.006.

[desc70217-bib-0101] Veijonaho, S. , L. Hietajärvi , M. Ojala , and K. Salmela‐Aro . 2025. “From Distress to Action? A Three‐Wave Longitudinal Study of Climate Change Distress, Pro‐Environmental Behavior, and Coping Strategies Among Finnish Adolescents.” Journal of Environmental Psychology 105: 102676. 10.1016/j.jenvp.2025.102676.

[desc70217-bib-0102] Veijonaho, S. , M. Ojala , L. Hietajärvi , and K. Salmela‐Aro . 2024. “Profiles of Climate Change Distress and Climate Denialism During Adolescence: A Two‐Cohort Longitudinal Study.” International Journal of Behavioral Development 48, no. 2: 103–112. 10.1177/01650254231205251.

[desc70217-bib-0103] Vlasceanu, M. , K. C. Doell , J. B. Bak‐Coleman , et al. 2024. “Addressing Climate Change With Behavioral Science: A Global Intervention Tournament in 63 Countries.” Science Advances 10, no. 6: eadj5778. 38324680 10.1126/sciadv.adj5778 PMC10849597

[desc70217-bib-0104] Vu, T. , L. Magis‐Weinberg , B. R. Jansen , et al. 2022. “Motivation‐Achievement Cycles in Learning: A Literature Review and Research Agenda.” Educational Psychology Review 34, no. 1: 39–71.

[desc70217-bib-0105] Wahlström M. , Wennerhag M. , Vydt M. D. , and Moor J. , eds. 2020. Protest for a Future—Composition, Mobilization and Motives of the Participants in Fridays for Future Climate Protests On 20‐27 September, 2019, in 19 Cities Around the World . https://www.diva‐portal.org/smash/get/diva2:1397070/FULLTEXT01.pdf.

[desc70217-bib-0106] Wanous, J. P. , A. E. Reichers , and M. J. Hudy . 1997. “Overall Job Satisfaction: How Good Are Single‐Item Measures?.” Journal of Applied Psychology 82, no. 2: 247. 9109282 10.1037/0021-9010.82.2.247

[desc70217-bib-0107] Wald, F. D. , and G. J. Mellenbergh . 1990. “De Verkorte Versie Van De Nederlandse Vertaling Van De Profile of Mood States (POMS).” *Nederlands Tijdschrift voor de Psychologie en haar Grensgebieden* . 5978910

[desc70217-bib-0108] Witte, K. 1992. “Putting the Fear Back Into Fear Appeals: the Extended Parallel Process Model.” Communications Monographs 59, no. 4: 329–349.

[desc70217-bib-0109] Witte, K. , and M. Allen . 2000. “A Meta‐Analysis of Fear Appeals: Implications for Effective Public Health Campaigns.” Health Education & Behavior 27, no. 5: 591–615. 11009129 10.1177/109019810002700506

[desc70217-bib-0110] Wullenkord, M. C. , M. Ojala , and S. Berger . 2023. “Climate‐Change Worry Among Two Cohorts of Late Adolescents: Exploring Macro and Micro Worries, Coping, and Relations to Climate Engagement, Pessimism, and Well‐being.” Journal of Environmental Psychology 90: 102093. 10.1016/j.jenvp.2023.102093.

[desc70217-bib-0111] Wullenkord, M. C. , J. Schulz , and S. M. Geiger . 2025. “Climate Anxiety—Impairment and/or Activation? Exploring the Roles of Mindfulness and Emotion Regulation.” Journal of Environmental Psychology 105: 102664. 10.1016/j.jenvp.2025.102664.

[desc70217-bib-0112] Yeager, D. S. , R. E. Dahl , and C. S. Dweck . 2018. “Why Interventions to Influence Adolescent Behavior Often Fail but Could Succeed.” Perspectives on Psychological Science 13, no. 1: 101–122. 29232535 10.1177/1745691617722620 PMC5758430

