# Supplementary material for: Youth Perspectives on the Climate Crisis: Motivation and Action Pathways
Source: Dev Sci. 2026 May 15;29:e70217. doi: 10.1111/desc.70217 (PMC13178203; doi:10.1111/desc.70217)
Supplement: Supplementary file 1 — Supporting File 1: desc70217‐sup‐0001‐SuppMat.docx. [file DESC-29-e70217-s001.docx]

**Supporting Information**

**Supporting Information Overview**

The following supplementary analyses were conducted to assess the robustness of our main findings. Specifically, we explored whether key relationships varied across age groups (below vs. above 18 years), providing sensitivity analyses for:

- Predictors of pro-environmental behavior and interactions with motivation type
- Motivation type
- Climate communication preferences
- Topics and survey findings presented across youth-panel sessions
- Self-designed pro-environmental behavior questionnaire

These analyses help to contextualize the developmental relevance of our findings and determine whether educational and psychological interventions should be age-tailored.

**Sensitivity Analyses – Pro-environmental Behavior and Motivation Type**

Although we found no age effects in any of the models, we decided to explore whether the relationships between motivation type and pro-environmental behavior might be different for adolescents under the age of 18 versus adolescents/young adults over the age of 18.

*Under 18 (n = 169)*In a multiple regression analysis with pro-environmental behavior as dependent variable and interactions between climate-related worry and each motivation type as predictors, including age, gender, education level, and cultural background as covariates, identified motivation significantly predicted greater pro-environmental behavior (*β* = .49, *t* = 3.02, *p* = .003). Climate-related worry change showed no main effect (*β* = –.11, *p* = .32), but interacted significantly with intrinsic motivation (*β* = .31, *t* = 2.24, *p* = .027) and identified motivation (*β* = –.32, *t* = –2.21, *p* = .029). The interaction with extrinsic motivation was not significant (*β* = –.16, *p* = .073). In adolescents under 18, identified motivation was the strongest predictor of pro-environmental behavior. Climate-related worry enhanced behavior in those high in intrinsic motivation, but reduced behavior among those high in identified motivation.

*Over 18 (n = 158)*In a multiple regression analysis with pro-environmental behavior as dependent variable and interactions between climate-related worry and each motivation type as predictors, including age, gender, education level, and cultural background as covariates, identified motivation again significantly predicted greater pro-environmental behavior (*β* = .61, *t* = 4.60, *p* < .001). Climate-related worry showed no main effect (*β* = –.02, *p* = .82), but interacted significantly with introjected motivation (*β* = .23, *t* = 2.61, *p* = .010). The interactions with intrinsic motivation (*β* = .16, *t* = 1.69, *p* = .094) and identified motivation (*β* = –.22, *t* = –1.76, *p* = .081) were not significant. Cultural background (non-Dutch monocultural) was negatively associated with pro-environmental behavior (*β* = –1.27, *t* = –2.65, *p* = .009). Among older participants, identified motivation remained a strong driver of climate behavior. Climate-related worry was only significantly related to behavior in those with higher introjected motivation.

**Sensitivity analyses – Motivation Types**

To explore differences in types of motivation to engage in pro-environmental behavior, we conducted pairwise comparisons across all participants using Bonferroni-corrected *t*-tests.

Supplementary Table 1 and Supplementary Figure 2 show that among adolescents *under 18*, identified motivation stood out as the most strongly endorsed type, suggesting that environmentally friendly behavior in this group is primarily driven by personally held values.

A similar pattern was observed among participants *above 18*, though average scores were generally slightly higher across all motivation types, suggesting greater internalization of pro-environmental values with age. Overall, identified motivation was rated higher than all other motivation types in the full sample, reinforcing its central role in driving climate behavior in youth. The consistently low endorsement of extrinsic motivation highlights that external pressure alone is not an effective motivator for climate action among adolescents or young adults.

**Supplementary Table 1***Pairwise comparisons between motivation types across ages*

| **Motivation 1** | **Motivation 2** | **Age** | **t-value** | **df** | **p-value** | **p (adj)** | **Cohen's d** |
| --- | --- | --- | --- | --- | --- | --- | --- |
| identified | extrinsic | All | 15.610 | 326.0 | <.001 | <.001 | 0.860 |
| identified | introjected | All | 12.670 | 326.0 | <.001 | <.001 | 0.700 |
| intrinsic | extrinsic | All | 9.360 | 326.0 | <.001 | <.001 | 0.520 |
| intrinsic | identified | All | -12.270 | 326.0 | <.001 | <.001 | -0.680 |
| intrinsic | introjected | All | 3.170 | 326.0 | .002 | .030 | 0.180 |
| introjected | extrinsic | All | 9.070 | 326.0 | <.001 | <.001 | 0.500 |
| identified | extrinsic | <18 | 9.240 | 122.0 | <.001 | <.001 | 0.830 |
| identified | introjected | <18 | 8.980 | 122.0 | <.001 | <.001 | 0.810 |
| intrinsic | extrinsic | <18 | 6.830 | 122.0 | <.001 | <.001 | 0.620 |
| intrinsic | identified | <18 | -6.120 | 122.0 | <.001 | <.001 | -0.550 |
| intrinsic | introjected | <18 | 4.760 | 122.0 | <.001 | <.001 | 0.430 |
| introjected | extrinsic | <18 | 4.290 | 122.0 | <.001 | <.001 | 0.390 |
| identified | extrinsic | ≥18 | 12.570 | 203.0 | <.001 | <.001 | 0.880 |
| identified | introjected | ≥18 | 9.090 | 203.0 | <.001 | <.001 | 0.640 |
| intrinsic | extrinsic | ≥18 | 6.600 | 203.0 | <.001 | <.001 | 0.460 |
| intrinsic | identified | ≥18 | -10.810 | 203.0 | <.001 | <.001 | -0.760 |
| intrinsic | introjected | ≥18 | 0.570 | 203.0 | .567 | 1.000 | 0.040 |
| introjected | extrinsic | ≥18 | 8.120 | 203.0 | <.001 | <.001 | 0.570 |

*Note*: p_adj represents Bonferroni corrected p-values.

**Supplementary Figure 2**

*Type of motivation to engage in pro-environmental behavior by age group*


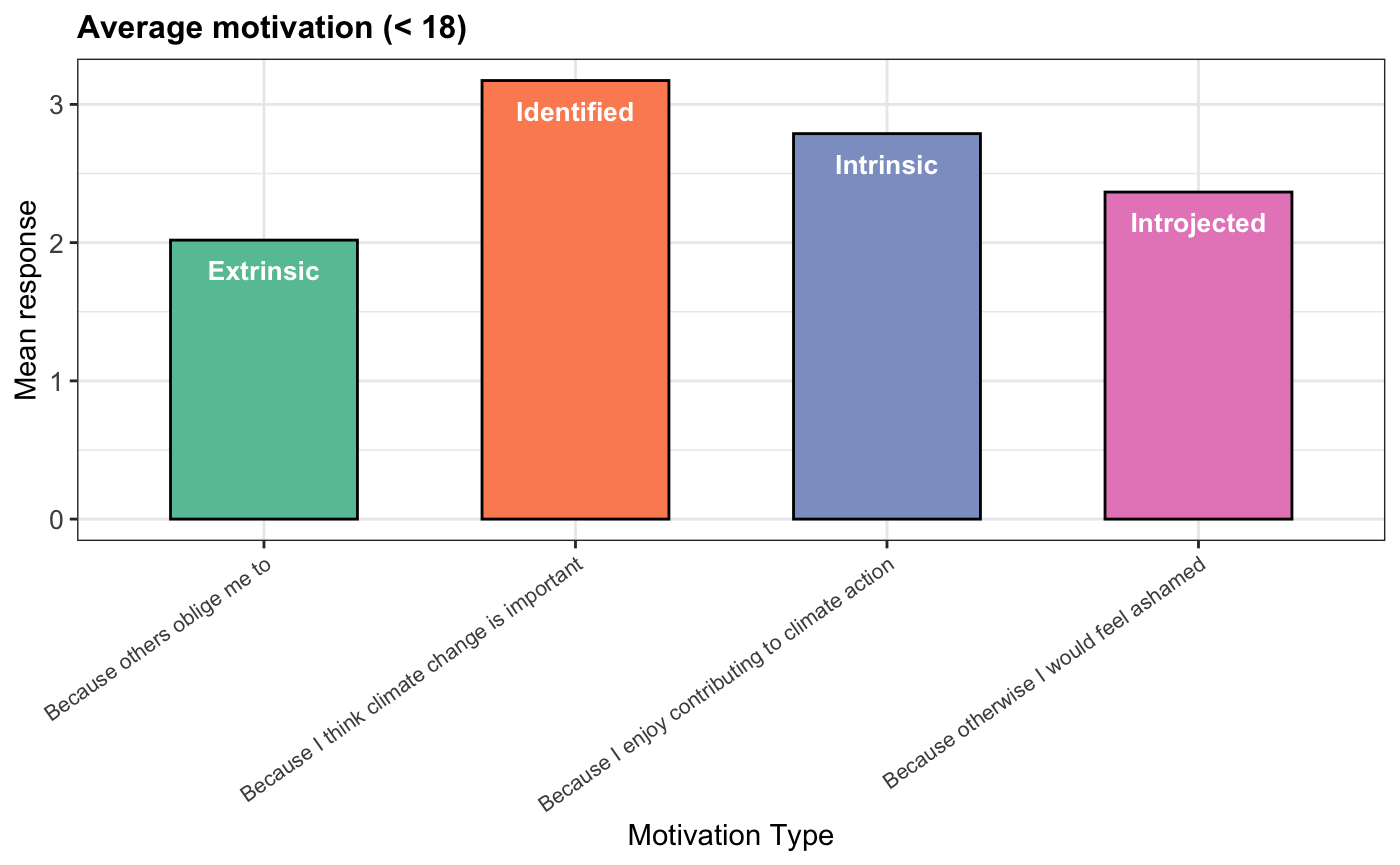


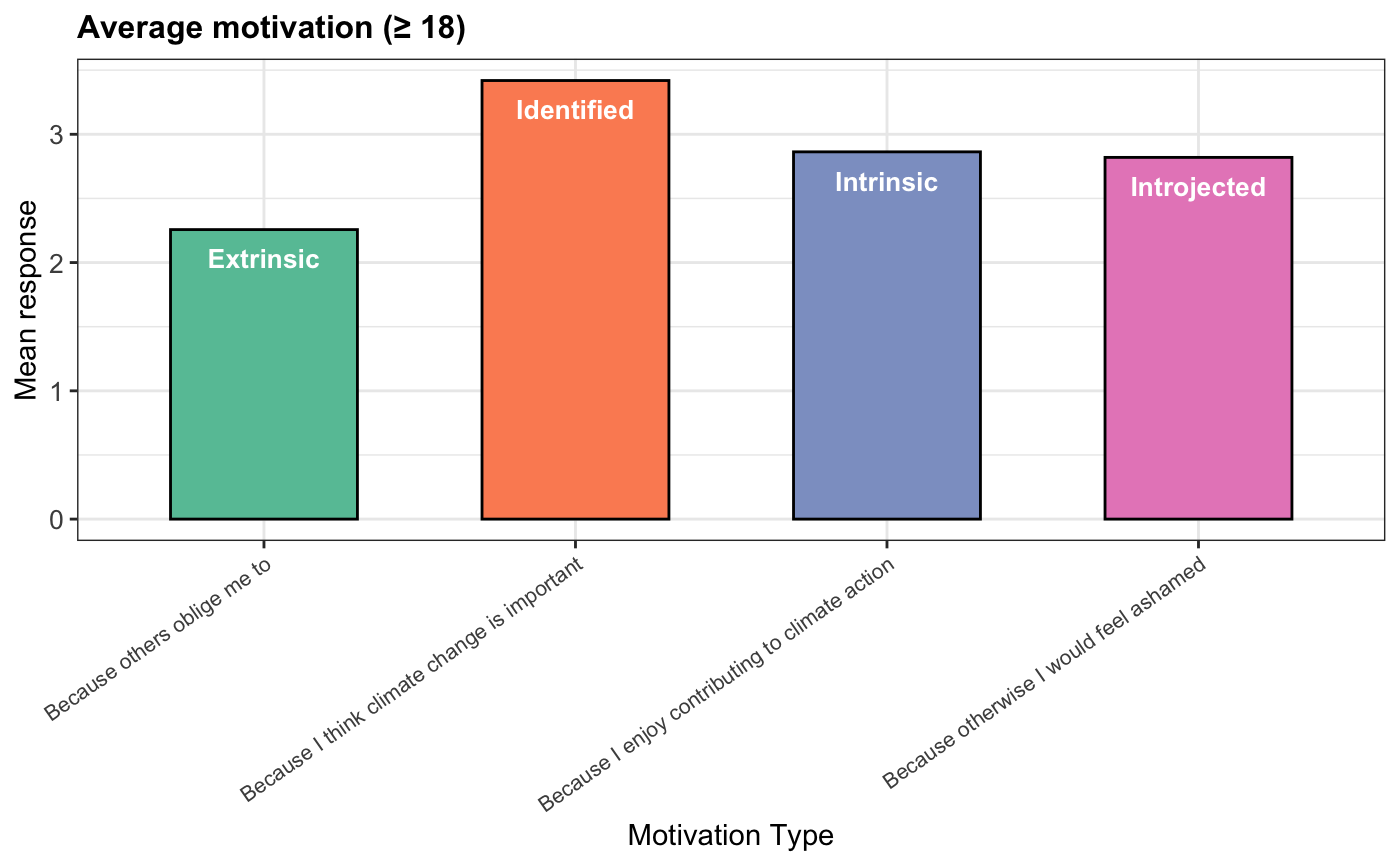


*Note*. X-axis labels display one illustrative item per motivation scale.

**Sensitivity Analyses – Communication preferences**

To examine age differences in climate communication preferences, we compared ratings across six strategies: Fear**,** Positive**,** Empowerment**,** Social norm**,** Information**,** and Humor. Supplementary Figure 3 shows the mean response per strategy for participants under and over 18 and Supplementary Table 2 shows pairwise comparisons across age groups. In both age groups, Fear-based messages received the highest ratings, while Humor and Information were rated lowest.

*Under 18 (n = 169)*Among adolescents under 18, Fear was rated significantly higher than Social norm (*p* < .001), but not significantly different from Empowerment (*p* = .221) or Positive (*p* = .557). Humor was rated significantly lower than Fear, Empowerment, and Positive (all *p* < .001), but did not differ from Information (*p* = 1.00) or Social norm (*p* = 1.00). Empowerment and Positive were rated equally (*p* = 1.00).

*Over 18 (n = 158)*
Among participants aged 18 and older, Fear was rated significantly higher than all other strategies (all *p* < .001). Humor was rated significantly lower than all others (all adjusted *p* ≤ .015). Empowerment and Positive again did not differ (*p* = 1.00), and Information was rated lower than Fear, Empowerment, and Positive (all adjusted *p* < .05), but not different from Social norm (*p* = 1.00).

**Supplementary Figure 3***Willingness to engage in pro-environmental behavior by communication strategy* *per age group*
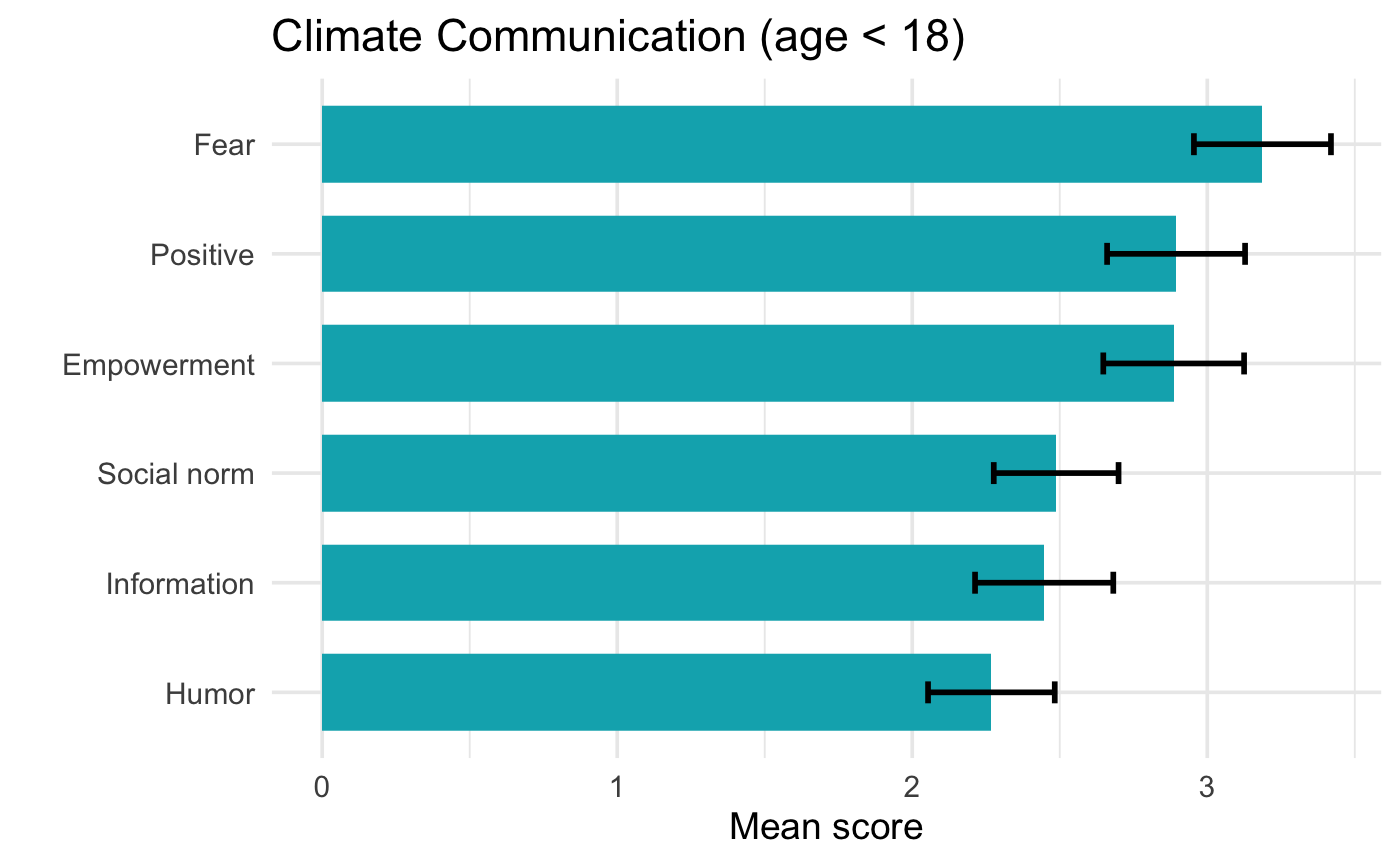


#### **
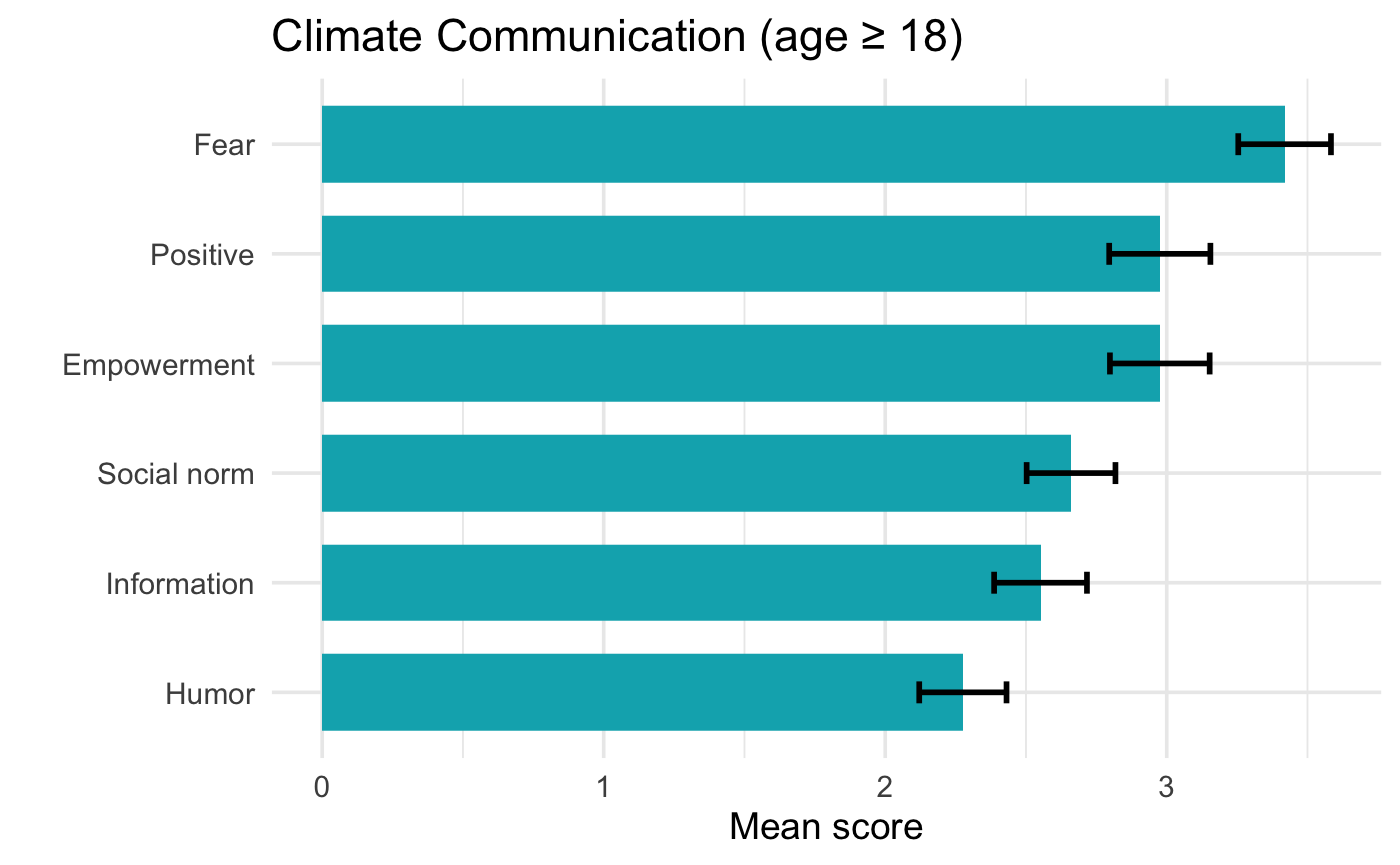
**

**Supplementary Table 2***Pairwise comparisons between communication strategies across age groups*

| **label1** | **label2** | **t** | **p** | **p_adj** | **Age group** | **Cohen’s d** |
| --- | --- | --- | --- | --- | --- | --- |
| Humor | Information | -3.398 | 0.001 | 0.015 | All | -0.188 |
| Humor | Fear | -13.971 | 0 | 0 | All | -0.774 |
| Humor | Social norm | -4.549 | 0 | 0 | All | -0.252 |
| Humor | Empowerment | -8.944 | 0 | 0 | All | -0.495 |
| Humor | Positive | -8.783 | 0 | 0 | All | -0.486 |
| Information | Fear | -11.448 | 0 | 0 | All | -0.634 |
| Information | Social norm | -1.124 | 0.262 | 1 | All | -0.62 |
| Information | Empowerment | -5.633 | 0 | 0 | All | -0.312 |
| Information | Positive | -5.247 | 0 | 0 | All | -0.291 |
| Fear | Social norm | 10.319 | 0 | 0 | All | 0.571 |
| Fear | Empowerment | 5.531 | 0 | 0 | All | 0.306 |
| Fear | Positive | 4.922 | 0 | 0 | All | 0.273 |
| Social norm | Empowerment | -5.729 | 0 | 0 | All | -0.317 |
| Social norm | Positive | -4.818 | 0 | 0 | All | -0.267 |
| Empowerment | Positive | -0.043 | 0.966 | 1 | All | -0.2 |
| Humor | Information | -1.402 | 0.163 | 1 | <18 | -0.126 |
| Humor | Fear | -7.307 | 0 | 0 | <18 | -0.659 |
| Humor | Social norm | -1.704 | 0.091 | 1 | <18 | -0.154 |
| Humor | Empowerment | -5.046 | 0 | 0 | <18 | -0.455 |
| Humor | Positive | -4.749 | 0 | 0 | <18 | -0.428 |
| Information | Fear | -6.35 | 0 | 0 | <18 | -0.573 |
| Information | Social norm | -0.316 | 0.753 | 1 | <18 | -0.28 |
| Information | Empowerment | -3.413 | 0.001 | 0.015 | <18 | -0.308 |
| Information | Positive | -3.203 | 0.002 | 0.03 | <18 | -0.289 |
| Fear | Social norm | 5.601 | 0 | 0 | <18 | 0.505 |
| Fear | Empowerment | 2.474 | 0.015 | 0.225 | <18 | 0.223 |
| Fear | Positive | 2.107 | 0.037 | 0.555 | <18 | 0.19 |
| Social norm | Empowerment | -4.193 | 0 | 0 | <18 | -0.378 |
| Social norm | Positive | -3.315 | 0.001 | 0.015 | <18 | -0.299 |
| Empowerment | Positive | -0.071 | 0.944 | 1 | <18 | -0.6 |
| Humor | Information | -3.335 | 0.001 | 0.015 | ≥18 | -0.234 |
| Humor | Fear | -12.083 | 0 | 0 | ≥18 | -0.848 |
| Humor | Social norm | -4.65 | 0 | 0 | ≥18 | -0.326 |
| Humor | Empowerment | -7.397 | 0 | 0 | ≥18 | -0.519 |
| Humor | Positive | -7.479 | 0 | 0 | ≥18 | -0.525 |
| Information | Fear | -9.558 | 0 | 0 | ≥18 | -0.671 |
| Information | Social norm | -1.216 | 0.225 | 1 | ≥18 | -0.85 |
| Information | Empowerment | -4.475 | 0 | 0 | ≥18 | -0.314 |
| Information | Positive | -4.149 | 0 | 0 | ≥18 | -0.291 |
| Fear | Social norm | 8.794 | 0 | 0 | ≥18 | 0.617 |
| Fear | Empowerment | 5.165 | 0 | 0 | ≥18 | 0.363 |
| Fear | Positive | 4.718 | 0 | 0 | ≥18 | 0.331 |
| Social norm | Empowerment | -4.023 | 0 | 0 | ≥18 | -0.282 |
| Social norm | Positive | -3.505 | 0.001 | 0.015 | ≥18 | -0.246 |
| Empowerment | Positive | 0 | 1 | 1 | ≥18 | 0.00 |

Note: p_adj represents Bonferroni corrected p-values.

**Supplementary Table 3.**

*Topic and survey findings presented in youth-panel sessions.*

| **Survey findings presented (i.e., ‘facts’)** |
| --- |
| 75 out of 100 youth see climate change as a problem. 79% finds it important that climate change is addressed. 82% knows climate change is a serious problem, but 54.8% among MBO students. |
| 68% worries much to very much about the climate. About half of youth reports stress about the future due to climate worries. |
| The more worries youth have, the more they also engage in pro environmental actions. 72% is motivated to contribute because they find it personally important. 37% would do something when it is imposed. |
| 81% thinks they as an individual can contribute. 90% thinks we together can really do something about climate change. 76% prefers large societal efforts to tackle climate change. |
| Youth describe responsibility as moving from collective to individual actions, rules for companies through to buying fewer things. Perception gap: youth rate it 75 out of 100 as a problem, but think parents and friends rate it 65 out of 100. |
| 90% thinks collective action can really work (collective efficacy anchor). 76% prefers large societal efforts. |
| 46% reports sometimes doing things that help the climate. 27% reports often or very often doing things that help the climate. |
| 89% talks about climate and sustainable behavior with others. 32% says parents and 21% says friends made more sustainable choices because of conversations with them. |
| Fear based communication followed by positive or empowering messaging works best for action willingness. |
| Youth talk about climate with parents (63.9%) and friends (66.1%) but less so at school (39.3%). Half of youth thinks they do not have the skills needed to deal with climate problems. |
| 76% prefers large societal efforts, and youth frame responsibility from collective to individual actions. |

**Supplementary Table 4.***Pro-environmental motivation questionnaire translated from SRQ-A.*

| **Motive** | **Original items self-regulation questionnaire academic (Vansteenkiste et al., 2009)** | **Climate adapted items (English)** | **Climate adapted items (Dutch)** |
| --- | --- | --- | --- |
| Extrinsic | I do my schoolwork because other people (parents, friends, teachers) oblige me to. | I take action for the climate because other people (parents, friends, teachers) make me do it | Ik zet me in voor het klimaat omdat anderen (ouders, vrienden, leerkrachten) me ertoe dwingen. |
| Extrinsic | I do my schoolwork because other people (parents, friends, teachers) expect me to. | I take action for the climate because others (parents, friends, teachers) expect me to do this. | Ik zet me in voor het klimaat omdat anderen (ouders, vrienden, leerkrachten) verwachten dat ik dit doe. |
| Extrinsic | I do my schoolwork because others (parents, friends, teachers) would get angry if I didn’t. | I take action for the climate because others (parents, friends, teachers) would get angry if I didn’t. | Ik zet me in voor het klimaat omdat anderen (ouders, vrienden, leerkrachten) boos zouden worden als ik het niet zou doen. |
| Extrinsic | I do my schoolwork because others (parents, friends, teachers) would be disappointed if I didn’t. | I take action for the climate because others (parents, friends, teachers) would be disappointed if I didn’t. | Ik zet me in voor het klimaat omdat anderen (ouders, vrienden, leerkrachten) teleurgesteld zouden zijn als ik het niet zou doen. |
| Introjected | I do my schoolwork because I want others to think I am a good student. | I take action for the climate because I want others to think that I take action for the climate. | Ik zet me in voor het klimaat omdat ik wil dat anderen denken dat ik me inzet voor het klimaat. |
| Introjected | I do my schoolwork because I would feel guilty if I didn’t. | I take action for the climate because I would feel guilty if I didn’t. | Ik zet me in voor het klimaat omdat ik me schuldig zou voelen als ik het niet zou doen. |
| Introjected | I do my schoolwork because I would feel ashamed if I didn’t. | I take action for the climate because I would feel ashamed if I didn’t. | Ik zet me in voor het klimaat omdat ik me zou schamen als ik het niet zou doen. |
| Introjected | I do my schoolwork because I want to give others the impression that I work hard at school. | I take action for the climate because I want to give others the impression that I take action for the climate. | Ik zet me in voor het klimaat omdat ik anderen de indruk wil geven dat ik me inzet voor het klimaat. |
| Identified | I do my schoolwork because I want to contribute to a better future for myself. | I take action for the climate because I want to contribute to a better climate. | Ik zet me in voor het klimaat omdat ik graag wil bijdragen aan een beter klimaat. |
| Identified | I do my schoolwork because it is personally very important to me. | I take action for the climate because I find this personally very important. | Ik zet me in voor het klimaat omdat ik dit persoonlijk heel belangrijk vind. |
| Identified | I do my schoolwork because it really matters to me personally. | I take action for the climate because this is personally important to me. | Ik zet me in voor het klimaat omdat dit voor mij persoonlijk belangrijk is. |
| Identified | I do my schoolwork because I see it as an important life goal. | I take action for the climate because I see this as an important life goal. | Ik zet me in voor het klimaat omdat ik dit een belangrijk levensdoel vind. |
| Intrinsic | I do my schoolwork because I find it interesting. | I take action for the climate because the climate really interests me. | Ik zet me in voor het klimaat omdat het klimaat mij erg interesseert. |
| Intrinsic | I do my schoolwork because I enjoy it. | I take action for the climate because taking action for the climate is fun. | Ik zet me in voor het klimaat omdat je inzetten voor het klimaat leuk is. |
| Intrinsic | I do my schoolwork because I find it engaging. | I take action for the climate because I find taking action for the climate engaging. | Ik zet me in voor het klimaat omdat ik me inzetten voor het klimaat boeiend vind. |
| Intrinsic | I do my schoolwork because I find it pleasant. | I take action for the climate because I find taking action for the climate enjoyable. | Ik zet me in voor het klimaat omdat ik me inzetten voor het klimaat aangenaam vind. |
